# Peroxiredoxin Asp f3 Is Essential for Aspergillus fumigatus To Overcome Iron Limitation during Infection

**DOI:** 10.1128/mBio.00976-21

**Published:** 2021-08-17

**Authors:** Victor Brantl, Jana M. Boysen, Annie Yap, Evgeny Golubtsov, Dominik Ruf, Thorsten Heinekamp, Maria Straßburger, Karl Dichtl, Hubertus Haas, Falk Hillmann, Johannes Wagener

**Affiliations:** a Max von Pettenkofer-Institut für Hygiene und Medizinische Mikrobiologie, Medizinische Fakultät, LMU München, Munich, Germany; b Evolution of Microbial Interactions, Leibniz Institute for Natural Product Research and Infection Biology—Hans Knöll Institute, Jena, Germany; c Institute of Microbiology, Friedrich Schiller University Jena, Jena, Germany; d Institute of Molecular Biology, Medical University of Innsbruck, Innsbruck, Austria; e Institut für Hygiene und Mikrobiologie, Julius-Maximilians-Universität Würzburg, Würzburg, Germany; f Department of Molecular and Applied Microbiology, Leibniz Institute for Natural Product Research and Infection Biology—Hans Knöll Institute, Jena, Germany; g Transfer Group Anti-infectives, Leibniz Institute for Natural Product Research and Infection Biology—Hans Knöll Institute, Jena, Germany; h Department of Clinical Microbiology, School of Medicine, Trinity College Dublin, The University of Dublin, St James’s Hospital Campus, Dublin, Ireland; IMBB-FORTH

**Keywords:** Asp f3, *Aspergillus fumigatus*, peroxiredoxin, iron regulation, virulence

## Abstract

Aspergillus fumigatus is an important fungal pathogen that causes allergic reactions but also life-threatening infections. One of the most abundant A. fumigatus proteins is Asp f3. This peroxiredoxin is a major fungal allergen and known for its role as a virulence factor, vaccine candidate, and scavenger of reactive oxygen species. Based on the hypothesis that Asp f3 protects A. fumigatus against killing by immune cells, we investigated the susceptibility of a conditional *aspf3* mutant by employing a novel assay. Surprisingly, Asp f3-depleted hyphae were killed as efficiently as the wild type by human granulocytes. However, we identified an unexpected growth defect of mutants that lack Asp f3 under low-iron conditions, which explains the avirulence of the Δ*aspf3* deletion mutant in a murine infection model. A. fumigatus encodes two Asp f3 homologues which we named Af3l (Asp f3-like) 1 and Af3l2. Inactivation of Af3l1, but not of Af3l2, exacerbated the growth defect of the conditional *aspf3* mutant under iron limitation, which ultimately led to death of the double mutant. Inactivation of the iron acquisition repressor SreA partially compensated for loss of Asp f3 and Af3l1. However, Asp f3 was not required for maintaining iron homeostasis or siderophore biosynthesis. Instead, we show that it compensates for a loss of iron-dependent antioxidant enzymes. Iron supplementation restored the virulence of the Δ*aspf3* deletion mutant in a murine infection model. Our results unveil the crucial importance of Asp f3 to overcome nutritional immunity and reveal a new biological role of peroxiredoxins in adaptation to iron limitation.

## INTRODUCTION

Several molds in the genus Aspergillus are important opportunistic fungal pathogens that cause a broad range of human diseases (1). These include severe invasive infections in immunocompromised patients, so-called invasive aspergillosis (IA; mortality, approximately 30 to 95%), chronic noninvasive (aspergilloma) or semi-invasive colonization of body cavities in healthy or generally immunocompetent individuals, and allergic diseases such as allergic bronchopulmonary aspergillosis (ABPA) and asthma (2–4). In most cases, Aspergillus fumigatus is the causative agent (1, 4).

Neutrophils play a major role in the defense against IA (1, 4, 5). Because of this, IA occurs almost exclusively in patients with primary or secondary immunodeficiencies that correlate with neutrophil dysfunction (2, 3). In contrast, allergic diseases result from the exuberant response of the immune system to allergens. Continuing inhalation of Aspergillus conidia and hyphal fragments or transient or chronic colonization of the respiratory tract by aspergilli may cause a hypersensitive immune reaction in atopic individuals. This hypersensitivity is typically characterized by IgE and IgG antibodies directed against Aspergillus antigens (6, 7). Asp f3 is a highly abundant protein in A. fumigatus and was identified, among others, as a major fungal allergen (8, 9). Cutaneous hypersensitivity or positive antibodies in serum against Aspergillus antigens, including Asp f3, indicate sensitization, which was proposed as obligatory criterion for diagnosing ABPA (10).

The cellular role of Asp f3 in A. fumigatus remained unknown for a long time. In a proteomic approach, Lessing and colleagues showed that Asp f3 is the most upregulated protein in A. fumigatus after exposure to hydrogen peroxide (11). Deletion of the encoding gene, *aspf3*, results in complete loss of virulence in a murine infection model (12). Based on its amino acid sequence, Asp f3 is a peroxiredoxin. This biochemical function was later demonstrated with crude extracts of Aspergillus in an enzymatic assay (12). The Δ*aspf3* deletion mutant is highly susceptible to hydrogen peroxide and the organic hydroperoxide *tert*-butyl hydroperoxide (*t*-bOOH) (12). It was therefore suggested that Asp f3 has an important function in peroxide detoxification and thereby contributes to virulence, making the pathogen more resistant to reactive oxygen species (ROS) which are released by innate immune cells (e.g., granulocytes) to counter the infection (11, 12). Interestingly, Asp f3 was additionally proposed as a vaccine candidate against IA, since Asp f3-primed CD4^+^ T cells can protect immunosuppressed mice from experimentally induced pulmonary aspergillosis (13, 14).

We recently established a novel killing assay to quantify the antifungal activity of granulocytes (15). In the present study, we used this assay to study the role of Asp f3 for Aspergillus to withstand killing by human granulocytes. Unexpectedly, we found that an Asp f3-depleted mutant is not more susceptible to killing than the wild type. In contrast, we detected a severe growth phenotype of Asp f3-lacking mutants under low-iron conditions. Furthermore, we identified an additional peroxiredoxin with partial functional redundancy, which we named Af3l1 (Asp f3-like 1). Importantly, supplementation with iron restored virulence of the Δ*aspf3* deletion mutant in a murine infection model. Based on our results, we highlight an unexpected key role of Asp f3 and its partially redundant homologue Af3l1 to overcome nutritional immunity during infection. In addition, we uncover a new biological role of peroxiredoxins.

## RESULTS

### Asp f3-depleted hyphae are H_2_O_2_ sensitive but not more efficiently killed by human granulocytes.

**FIG 1:**
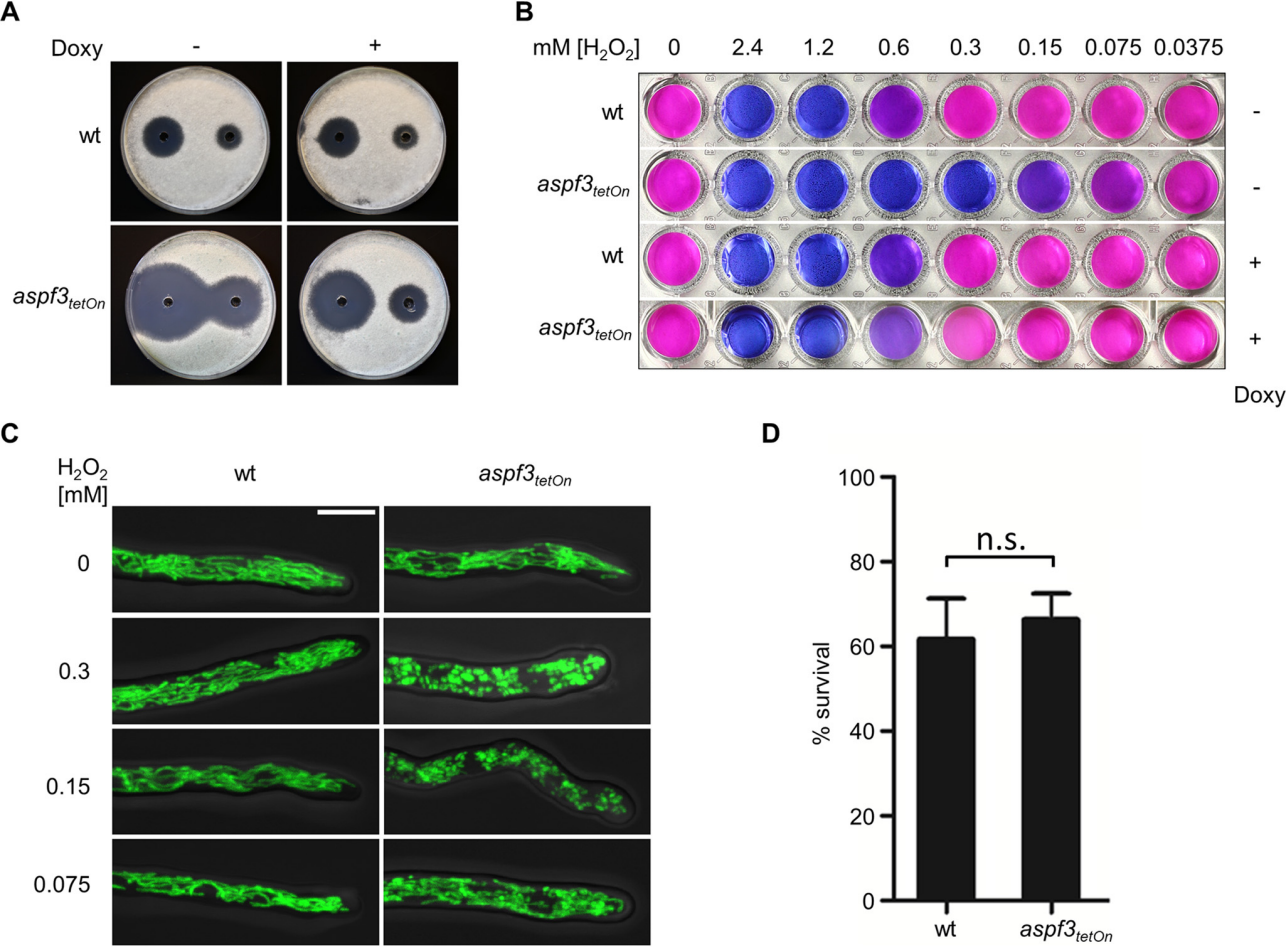
An Asp f3-depleted mutant is hypersensitive to hydrogen peroxide but is not more effectively killed by human granulocytes. (A) Conidia (4 × 10^5^) of the indicated strains were spread on AMM agar plates. When indicated, medium was supplemented with doxycycline (7.5 μg ml^−1^; Doxy). Fifty microliters of 300 mM (left) or 100 mM (right) H_2_O_2_ was applied in the punch holes of each agar plate. Images were taken after 30 h of incubation at 37°C. (B) Conidia (1.5 × 10^4^) of the indicated strains were inoculated in 100 μl RPMI 1640 per well in a 96-well plate and incubated at 37°C with 5% CO_2_. After 10 h, 100 μl medium supplemented with resazurin and, when indicated, H_2_O_2_ was added to a final concentration of 0.002% (wt/vol) resazurin and the indicated final concentration of H_2_O_2_. The plate was then incubated for another 24 h at 37°C with 5% CO_2_. (C) Conidia of the indicated strains expressing mitochondrion-targeted GFP (mtGFP) were inoculated in RPMI 1640 and incubated at 37°C with 5% CO_2_. After 10 h, medium was supplemented with the indicated H_2_O_2_ concentrations. After 2 h of incubation, samples were fixed and analyzed with a confocal laser scanning microscope. The depicted representative images are overlays of bright-field and GFP fluorescence images of optical stacks covering the entire hypha in focus. Bar, 5 μm (applicable to all images). (D) Conidia of the indicated strains expressing mtGFP were inoculated in RPMI 1640. Human granulocytes (PMNs) were added after 10 h of incubation at 37°C with 5% CO_2_. After 2 h coincubation (37°C, 5% CO_2_), samples were fixed and stained with calcofluor white. The ratio of vital hyphae (defined as hyphae with tubular or partially tubular mitochondrial morphology in more than 40% of a hyphal volume) was determined as described in Materials and Methods. The data in the graph are based on the results of three independent experiments. Statistical significance (n.s., not significant [*P* > 0.05]) was calculated with a two-tailed unpaired (assuming unequal variances) Student’s *t* test. The error bars indicate standard deviations.

ROS are of central importance for the host defense against invasive Aspergillus infections. This is evidenced by that fact that patients with chronic granulomatous disease (CGD), a hereditary inability of immune cells to produce various ROS, are at high risk for invasive aspergillosis (16). However, it remains controversial whether ROS directly kill the pathogens or indirectly mediate the immune defense, e.g., by promoting neutrophil degranulation and extracellular trap formation (5). The Δ*aspf3* deletion mutant is one of very few A. fumigatus mutants described that showed both increased susceptibility to ROS and avirulence in a murine infection model (12). To study the role of human granulocytes in inactivating A. fumigatus that lacks the peroxide detoxification enzyme Asp f3, we constructed a conditional *aspf3* mutant by replacing the endogenous promoter with a doxycycline-inducible Tet-On promoter system (*aspf3_tetOn_*). Growth of the wild type and of the induced or repressed *aspf3_tetOn_* mutant was indistinguishable with respect to germination, growth rate, and formation of conidia (asexual spores) under normal growth conditions (data not shown). However, under repressed conditions, the conditional *aspf3_tetOn_* mutant exhibited a severe susceptibility to hydrogen peroxide on solid agar and in liquid medium (Fig. 1A and B), very similar to a Δ*aspf3* deletion mutant characterized in previous studies. Induction of the conditional promoter partially rescued the increased hydrogen peroxide susceptibility of the mutant (Fig. 1A and B).

To assess the susceptibility of the *aspf3_tetOn_* mutant to killing by human granulocytes, we constructed a derivative that constitutively expresses mitochondrion-targeted green fluorescent protein (mtGFP). This reporter, which we called MitoFLARE, allows visualizing and quantifying of hydrogen peroxide- as well as granulocyte-induced cell death of Aspergillus hyphae (15). Hydrogen peroxide readily induced fragmentation of the tubular mitochondrial network, thereby clearly indicating the increased ROS susceptibility of the conditional *aspf3_tetOn_* mutant under repressed conditions compared to the induced strain or to the mtGFP-expressing wild type (Fig. 1C). Next, we analyzed the susceptibility of the *aspf3_tetOn_* mutant to killing induced by granulocytes isolated from human blood (15). Surprisingly, the *aspf3_tetOn_* mutant under repressed conditions was not more prone to granulocyte-induced killing than the wild type (Fig. 1D).

### Unexpected growth phenotype under low-iron conditions.

**FIG 2:**
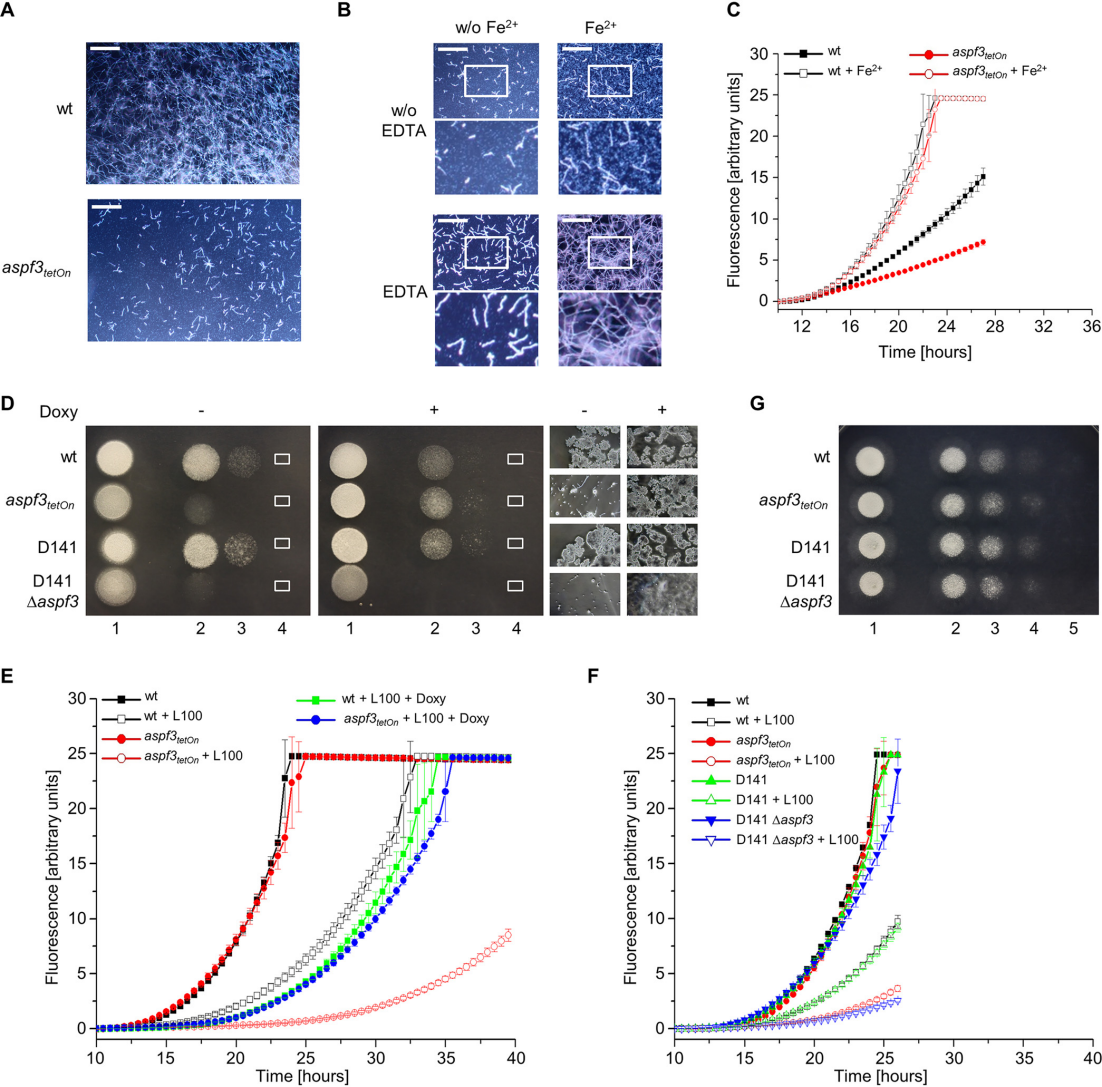
Asp f3 is important for growth under low-iron conditions. (A and B) Conidia (1.5 × 10^4^) of the indicated strains (A) or of the *aspf3_tetOn_* strain (B) were inoculated in RPMI 1640 medium supplemented with 0.002% (wt/vol) resazurin per well in a 96-well plate. When indicated, medium was additionally supplemented with or without (w/o) 5 μg ml^−1^ FeSO_4_ (Fe^2+^) or 50 μg ml^−1^ EDTA. Plates were incubated at 37°C at atmospheric CO_2_ concentration, causing partial precipitation of the medium. Representative dark-field images were taken after 10 h. Magnifications of the framed sections of the images are shown in the lower rows. Bars, 250 μm. (C, E, and F) Conidia (1.5 × 10^4^) of the indicated strains were inoculated in RPMI 1640 medium supplemented with 0.002% (wt/vol) resazurin per well in a 96-well plate. When indicated, medium was additionally supplemented with 100 ng ml^−1^ FeSO_4_ (Fe^2+^), 7.5 μg ml^−1^ doxycycline (Doxy), or 100 μg ml^−1^ lactoferrin (L100). The plate was then incubated at 37°C with 5% CO_2_. Resorufin fluorescence was documented over time with a microplate reader and plotted in the graphs. The error bars indicate standard deviations for three technical replicates. (D and G) In a series of 10-fold dilutions derived from a starting suspension of 5 × 10^7^ conidia ml^−1^ of the indicated strains, aliquots of 3 μl were spotted on peptone agarose plates (1% [wt/vol] agarose, 1% [wt/vol] peptone; pH 7.0) supplemented with 2.5 mg ml^−1^ lactoferrin. When indicated, medium was additionally supplemented with 7.5 μg ml^−1^ doxycycline (Doxy). The plate depicted in panel G was additionally supplemented with 5 μg ml^−1^ FeSO_4_. Representative images were taken after 30 h (D) or 24 h (G) incubation at 37°C. (D) Magnifications of the framed sections of the images are shown on the right.

Due to its drastically increased susceptibility to peroxides, we expected the conditional *aspf3_tetOn_* mutant under repressed conditions to be significantly more sensitive to killing by human granulocytes. We therefore attempted to confirm our result in an independent assay that relies on measuring the metabolic activity of the surviving fungi after killing by granulocytes with a colorimetric assay (e.g., see references 17–20). To this end, we inoculated conidia of the wild type and the *aspf3_tetOn_* strain under inducing and noninducing conditions in RPMI 1640 medium and incubated the respective well plates at 37°C overnight to obtain Aspergillus hyphae for exposing to granulocytes. Even though carbonate-buffered RPMI 1640 medium was used, the well plate was incubated at an atmospheric carbon dioxide concentration. This condition unveiled a growth defect (granulocyte independent) of the *aspf3_tetOn_* mutant under repressed conditions (Fig. 2A).

Apparently, the alkalization of the medium due to atmospheric carbon dioxide concentration and successive loss of the bicarbonate buffer caused precipitation of media components (Fig. 2A; also, see Fig. S1A in the supplemental material). We therefore tested which essential medium supplement can reconstitute growth of the Asp f3-depleted Aspergillus mutant. Of the three tested essential metals, i.e., iron, zinc, and copper, iron (Fe^2+^) showed a striking effect (Fig. S1B). Supplementation of the precipitated RPMI 1640 medium with iron sulfate chelated with EDTA (FeSO_4_-EDTA), which keeps iron soluble and bioavailable, apparently fully restored growth (Fig. 2B). To confirm these results, we artificially depleted media from free iron by adding bathophenanthrolinedisulfonic acid (BPS) or lactoferrin, a multifunctional protein with antibacterial and antifungal properties that binds iron. As shown in Fig. S1C and Fig. 2D and E, BPS and 2.5 mg ml^−1^ lactoferrin from bovine milk significantly inhibited growth of the *aspf3_tetOn_* mutant under repressed conditions. Very similar results were obtained for a Δ*aspf3* deletion mutant compared to its wild type (Fig. 2D and F). Doxycycline induced the conditional promoter of the *aspf3_tetOn_* mutant and fully rescued growth but did not affect the susceptibility of the Δ*aspf3* deletion mutant (Fig. 2D and E). Supplementation of the medium with iron, however, fully restored growth of both mutants, the repressed *aspf3_tetOn_* strain and the Δ*aspf3* strain (Fig. 2G and Fig. S1). This demonstrates that Asp f3 plays an important role in growth of A. fumigatus under low-iron conditions.

10.1128/mBio.00976-21.1 FIG S1 (A) RPMI 1640 supplemented with 0.002% (wt/vol) resazurin was incubated at 37°C and atmospheric CO_2_ concentration. Representative dark-field images were taken prior to incubation (top) and after approximately 10 h incubation (bottom), which caused partial precipitation of the medium. (B) Conidia (1.5 × 10^4^) of the indicated strains were inoculated in RPMI 1640 medium supplemented with 0.002% (wt/vol) resazurin per well in a 96-well plate. When indicated, medium was additionally supplemented with 5 μg ml^−1^ FeSO_4_, 1.6 μg ml^−1^ CuSO_4_, or 22 μg ml^−1^ ZnSO_4_ in 50 μg ml^−1^ EDTA. Plates were incubated at 37°C at atmospheric CO_2_ concentration, causing partial precipitation of the medium. Representative dark-field images were taken after approximately 24 h. (A and B) Bar, 250 μm (applicable to all images). (C) Conidia (1.5 × 10^4^) of the indicated strains were inoculated in 200 μl RPMI 1640 supplemented with 0.002% (wt/vol) resazurin and the indicated concentration of bathophenanthrolinedisulfonic acid (BPS) per well in a 96-well plate. The plate was incubated at 37°C with 5% CO_2_. After 66 h incubation, representative images were taken. Download FIG S1, PDF file, 0.3 MB. Copyright © 2021 Brantl et al. 2021 Brantl et al. https://creativecommons.org/licenses/by/4.0/ This content is distributed under the terms of the Creative Commons Attribution 4.0 International license.

### Identification of an Asp f3 homologue with functional overlap under low-iron but not under oxidative-stress conditions.

Asp f3 belongs to the atypical 2-Cys group of peroxiredoxins (12, 21, 22). BLAST searches revealed that A. fumigatus encodes two other Asp f3-like (Af3l) proteins, Afu5g01440 (Af3l1) and Afu6g12500 (Af3l2), both of which remained uncharacterized. All three proteins harbor a conserved redoxin (PF08534) protein family (Pfam) (23) pattern that is not found in any other A. fumigatus proteins. The opportunistic pathogenic yeast Candida albicans encodes three Asp f3/Af3l1/Af3l2 homologues (CaAhp1, CaAhp2, and CaTrp99) and baker’s yeast (Saccharomyces cerevisiae) only one (ScAhp1). Similar to A. fumigatus, other Aspergillus species, such as Aspergillus niger, Aspergillus flavus and Aspergillus nidulans, each encode three Asp f3/Af3l1/Af3l2 homologues. In all these species, the identified proteins are consistent with the proteins that harbor the conserved redoxin (PF08534) protein family pattern.

**FIG 3:**
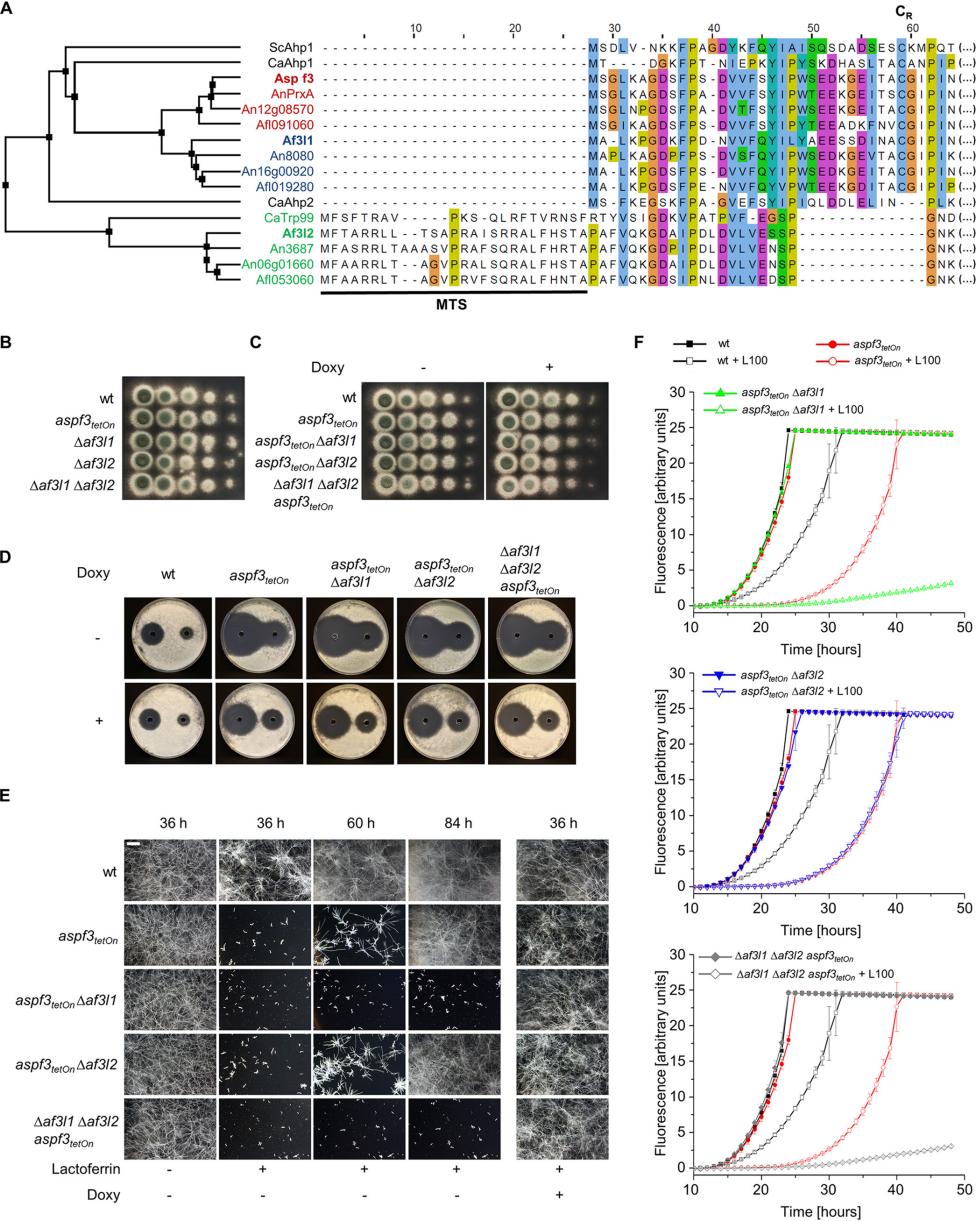
Identification and functional characterization of the Asp f3 homologues Af3l1 and Af3l2. (A) Average distance tree and alignment of the N-terminal sequences of Asp f3 and its homologues and putative paralogues in A. fumigatus (Af3l1 and Af3l2), A. nidulans (AnPrxA, An8080, and An3687), A. flavus (Afl091060, Afl019280, and Afl053060), A. niger (An12g08570, An16g00920, and An06g01660), S. cerevisiae (ScAhp1), and C. albicans (CaAhp1, CaAhp2, and CaTrp99). The black bar indicates a putative mitochondrial targeting signal (MTS). A conserved resolving cysteine (C_R_) is indicated. Sequences of Af3l1, Af3l2, Afl019280, and Afl053060 have been corrected taking into account most likely incorrectly annotated start codons or splice sites for the respective sequences in genome databases. Alignment (MAFFT, Clustal color scheme) is based on total protein sequences; the average distance tree was generated with BLOSUM62. The full alignment is available in Fig. S2. (B and C) In a series of 10-fold dilutions derived from a starting suspension of 5 × 10^7^ conidia ml^−1^ of the indicated strains, aliquots of 3 μl were spotted on AMM agar plates. When indicated, medium was supplemented with 7.5 μg ml^−1^ doxycycline (Doxy). Representative images were taken after 30 h of incubation at 37°C. (D) Conidia (4 × 10^5^) of the indicated strains were spread on AMM agar plates. When indicated, medium was supplemented with doxycycline (7.5 μg ml^−1^; Doxy). Fifty microliters of 300 mM (left) or 100 mM (right) H_2_O_2_ was applied in the punch holes of each agar plate. Images were taken after 30 h of incubation at 37°C. (E and F) Conidia (1.5 × 10^3^ [E] or 1.5 × 10^4^ [F]) of the indicated strains were inoculated in RPMI 1640 medium supplemented with 100 ng ml^−1^ FeSO_4_ per well in 96-well plates. For panel F, medium was additionally supplemented with 0.002% (wt/vol) resazurin. When indicated, medium was additionally supplemented with 100 μg ml^−1^ lactoferrin (L100) or 7.5 μg ml^−1^ doxycycline (Doxy). Plates were then incubated at 37°C with 5% CO_2_. (E) After the indicated incubation time, representative dark-field images were taken. Bar, 250 μm (applicable to all images). (F) Resorufin fluorescence was documented over time with a microplate reader and plotted in the graph. The error bars indicate standard deviations for three technical replicates.

Alignments of the identified predicted proteins and analysis of transcription start based on RNA sequencing data (24) revealed that the start sites of translation of Af3l1, Af3l2, and the A. flavus proteins encoded by AFLA_053060 and AFLA_019280 were most likely incorrectly annotated in the genome database (wrong start codon or splice sites). The corrected protein sequences, including full alignments thereof, are shown in Fig. S2. The tree depicted in Fig. 3A and Fig. S2 shows agglomerative clustering of the full alignments of Asp f3-like proteins based on average distance and indicates that Af3l1 and Asp f3 are more similar than Af3l2 and Asp f3. The A. nidulans Asp f3 homologue PrxA (AN8692) was recently characterized and is reported to have a function similar to that previously described for Asp f3 in A. fumigatus (25).

10.1128/mBio.00976-21.2 FIG S2 Average distance tree and full alignment of the sequences of Asp f3 and its homologues and putative paralogues in A. fumigatus (Af3l1 and Af3l2), A. nidulans (AnPrxA, An8080, and An3687), A. flavus (Afl091060, Afl019280, and Afl053060), A. niger (An12g08570, An16g00920, and An06g01660), S. cerevisiae (ScAhp1), and C. albicans (CaAhp1, CaAhp2, CaTrp99). The black bar indicates a putative mitochondrial targeting signal (MTS). The conserved resolving cysteine (C_R_), peroxidatic cysteine (C_P_), and arginine (*), which are characteristic of peroxiredoxins, are indicated. Sequences of Af3l1, Af3l2, Afl019280, and Afl053060 have been corrected taking into account most likely incorrectly annotated start codons or splice sites for the respective sequences in genome databases. Alignment (MAFFT; Clustal color scheme) is based on total protein sequences; the average distance tree was generated with BLOSUM62. Download FIG S2, PDF file, 0.6 MB. Copyright © 2021 Brantl et al. 2021 Brantl et al. https://creativecommons.org/licenses/by/4.0/ This content is distributed under the terms of the Creative Commons Attribution 4.0 International license.

In contrast to Asp f3, Af3l1 and related proteins, Af3l2 and its homologues (CaTrp99, An3687, and Afl053060) have an N-terminal extension of approximately 25 amino acids preceding the conserved redoxin domain (Fig. 3A). Analysis of the respective sequences with MitoFates (26) indicates that these are mitochondrial targeting signals. Af3l2 and its homologues are therefore most likely mitochondrial proteins. Interestingly, the Af3l2-like proteins lack the so-called “resolving” cysteine (C_R_) in the N-terminal part of the protein which is highly conserved in the Asp f3- and Af3l1-like proteins and in ScAhp1 as well as in CaAhp1 (Fig. S2). This cysteine is essential for forming homodimers and for the peroxidase activities of Asp f3 and AnPrxA (12, 25).

We asked whether Af3l1 and Af3l2 have functions similar to those of the peroxiredoxins Asp f3 or AnPrxA. We therefore constructed mutants that lack *af3l1* or *af3l2*. No growth phenotypes were found under standard growth conditions for both gene deletion mutants (Fig. 3B). Besides this, hydrogen peroxide susceptibility and growth rates under low-iron conditions of the Δ*af3l1* and Δ*af3l2* deletion mutants were similar to those of the wild type (Fig. S3A and B). To check for potential functional redundancy of the enzyme, we constructed double and triple mutants. As shown in Fig. 3C and D, deletion of *af3l1*, *af3l2*, or both did not significantly alter growth or the hydrogen peroxide susceptibility of the *aspf3_tetOn_* mutant under repressed conditions. Significant differences were observed under low-iron conditions. Growth of the *aspf3_tetOn_* Δ*af3l1* double mutant and of the Δ*af3l1* Δ*af3l2 aspf3_tetOn_* triple mutant under repressed conditions was drastically reduced compared to that of the *aspf3_tetOn_* single mutant in the presence of lactoferrin (Fig. 3E and F). Growth reduction under low-iron conditions of the *aspf3_tetOn_* Δ*af3l1* mutant and that of the Δ*af3l1* Δ*af3l2 aspf3_tetOn_* mutant were comparable. Deletion of *af3l2* did not change the low-iron susceptibility of the *aspf3_tetOn_* mutant under repressed conditions (Fig. 3E and F). Notably, the *aspf3_tetOn_* Δ*af3l1* and the Δ*af3l1* Δ*af3l2 aspf3_tetOn_* mutants were constructed independently but showed very similar increase of low-iron susceptibility under repressed conditions compared to the *aspf3_tetOn_* mutant (Fig. 3E and F). Induction of the Tet-On promoter restored growth of the conditional *aspf3_tetOn_* Δ*af3l1* mutant and the Δ*af3l1* Δ*af3l2 aspf3_tetOn_* mutant under low-iron conditions compared to the wild type (Fig. 3E and Fig. S4). This clearly demonstrates that Af3l1 is partially functionally redundant with Asp f3 under low-iron conditions.

10.1128/mBio.00976-21.3 FIG S3 (A) Conidia (4 × 10^5^) of the indicated strains were spread on AMM agar plates. When indicated, medium was supplemented with doxycycline (7.5 μg ml^−1^; Doxy). Fifty microliters of 300 mM (left) or 100 mM (right) H_2_O_2_ was applied in the punch holes of each agar plate. Images were taken after 30 h incubation at 37°C. (B) Conidia (1.5 × 10^4^) of the indicated strains were inoculated in RPMI 1640 medium supplemented with 0.002% (wt/vol) resazurin and 100 ng ml^−1^ FeSO_4_ per well in 96-well plates. When indicated, medium was additionally supplemented with 100 μg ml^−1^ lactoferrin. Plates were then incubated at 37°C with 5% CO_2_. Resorufin fluorescence was documented over time with a microplate reader and plotted in the graph. The error bars indicate standard deviations for three technical replicates. Download FIG S3, PDF file, 0.2 MB. Copyright © 2021 Brantl et al. 2021 Brantl et al. https://creativecommons.org/licenses/by/4.0/ This content is distributed under the terms of the Creative Commons Attribution 4.0 International license.

10.1128/mBio.00976-21.4 FIG S4 Conidia (1.5 × 10^4^) of the indicated strains were inoculated in RPMI 1640 medium supplemented with 0.002% (wt/vol) resazurin and 100 ng ml^−1^ FeSO_4_ per well in 96-well plates. When indicated, medium was additionally supplemented with 100 μg ml^−1^ lactoferrin (L100). Plates were then incubated at 37°C with 5% CO_2_. Resorufin fluorescence was documented over time with a microplate reader and plotted in the graph. The error bars indicate standard deviations for three technical replicates. Download FIG S4, PDF file, 0.2 MB. Copyright © 2021 Brantl et al. 2021 Brantl et al. https://creativecommons.org/licenses/by/4.0/ This content is distributed under the terms of the Creative Commons Attribution 4.0 International license.

### Asp f3 and Af3l1 are essential for survival under low-iron conditions.

**FIG 4:**
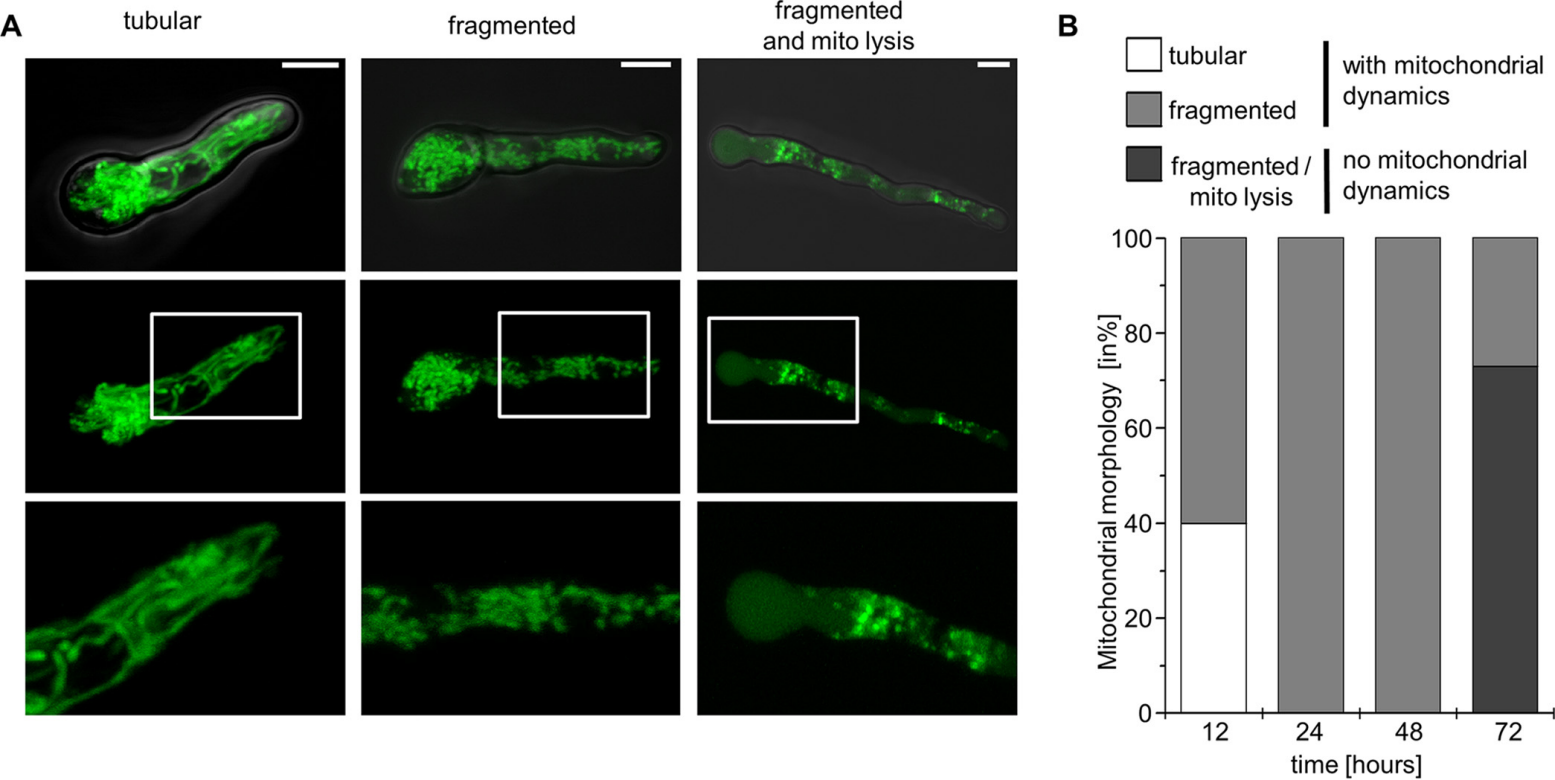
Asp f3 and Af3l1 are essential for survival under iron-limited conditions. (A and B) Conidia of the *aspf3_tetOn_* Δ*af3l1* mutant expressing mitochondrion-targeted GFP (mtGFP) were inoculated in RPMI 1640 supplemented with 100 ng ml^−1^ FeSO_4_ and 200 μg ml^−1^ lactoferrin under repressed conditions (no doxycycline). Samples were incubated at 37°C with 5% CO_2_ and then analyzed with a laser scanning microscope. (A) Representative GFP fluorescence images of hyphae with tubular (left) and fragmented (middle and right) mitochondrial morphology after 12 h, 24 h, and 72 h of incubation, respectively. The hypha on the right shows cytosolic fluorescence, which indicates disruption of mitochondrial integrity (mito lysis) and release of mtGFP to the cytosol. Depicted are images of optical stacks covering the entire hyphae in focus (middle row), magnifications of the framed sections therein (bottom row), and overlays of bright-field and GFP fluorescence images (top row). Bars, 5 μm. (B) The viability of 100 individual hyphae per time point was analyzed in independent samples after 12, 24, 48, and 72 h of incubation based on the mitochondrial morphology and dynamics. Hyphae which exhibited a tubular or fragmented mitochondrial morphology with mitochondrial dynamics were considered alive. Hyphae which exhibited a fragmented mitochondrial morphology without mitochondrial dynamics with or without cytosolic fluorescence or no fluorescence were considered dead.

Growth of the *aspf3_tetOn_* Δ*af3l1* double mutant under repressed and low-iron conditions was almost abolished. Nevertheless, the *aspf3_tetOn_* Δ*af3l1* conidia were overall able to germinate and form short hyphae, but hyphae then stopped growing (Fig. 3E and 4A). We asked whether these fungi die during germination. The *aspf3_tetOn_* Δ*af3l1* mutant was transformed with a construct expressing mtGFP to visualize mitochondria, in order to quantify viability of individual hyphae (27). Mitochondrial morphology and dynamics of the resulting strain were analyzed over time under repressed conditions (Fig. 4; Videos S1 to S3). As shown in Fig. 4, the germlings still showed dynamic mitochondrial morphology after 12, 24, and 48 h. After 72 h, approximately 70% of the hyphae showed clear evidence of cell death (lysis of mitochondria and release of mtGFP to the cytoplasm, arrest of mitochondrial dynamics, and fading of GFP fluorescence). This indicates that the *aspf3_tetOn_* Δ*af3l1* hyphae are unable to grow but remain alive for an extended period.

10.1128/mBio.00976-21.6 VIDEO S1 Exemplary video of an *aspf3_tetOn_* Δ*af3l1* mutant hypha after 12 h incubation with a dynamic tubular mitochondrial morphology. Conidia of the *aspf3_tetOn_* Δ*af3l1* mutant expressing mitochondrion-targeted GFP (mtGFP) were inoculated in RPMI 1640 supplemented with 100 ng ml^−1^ FeSO_4_ and 200 μg ml^−1^ lactoferrin under repressed conditions (no doxycycline). Samples were incubated at 37°C with 5% CO_2_ and analyzed with a laser scanning microscope over time. Short video sequences of optical stacks of the GFP fluorescence covering the entire hyphae in focus were recorded after 12 h. Download Movie S1, AVI file, 4.5 MB. Copyright © 2021 Brantl et al. 2021 Brantl et al. https://creativecommons.org/licenses/by/4.0/ This content is distributed under the terms of the Creative Commons Attribution 4.0 International license.

10.1128/mBio.00976-21.7 VIDEO S2 Exemplary videos of an *aspf3_tetOn_* Δ*af3l1* mutant hypha after 24 h incubation with fragmented, but still dynamic mitochondrial morphology. The video shows the hypha shown in Video S3 but after 24 h incubation. Conidia of the *aspf3_tetOn_* Δ*af3l1* mutant expressing mitochondrion-targeted GFP (mtGFP) were inoculated in RPMI 1640 supplemented with 100 ng ml^−1^ FeSO_4_ and 200 μg ml^−1^ lactoferrin under repressed conditions (no doxycycline). Samples were incubated at 37°C with 5% CO_2_ and analyzed with a laser scanning microscope over time. Short video sequences of optical stacks of the GFP fluorescence covering the entire hyphae in focus were recorded after 24 h. Download Movie S2, AVI file, 18.0 MB. Copyright © 2021 Brantl et al. 2021 Brantl et al. https://creativecommons.org/licenses/by/4.0/ This content is distributed under the terms of the Creative Commons Attribution 4.0 International license.

10.1128/mBio.00976-21.8 VIDEO S3 Exemplary videos of an *aspf3_tetOn_* Δ*af3l1* mutant hypha after 72 h incubation with fragmented nondynamic mitochondrial morphology. The video shows the hypha shown in Video 2 but after 72 h incubation. Conidia of the *aspf3_tetOn_* Δ*af3l1* mutant expressing mitochondrion-targeted GFP (mtGFP) were inoculated in RPMI 1640 supplemented with 100 ng ml^−1^ FeSO_4_ and 200 μg ml^−1^ lactoferrin under repressed conditions (no doxycycline). Samples were incubated at 37°C with 5% CO_2_ and analyzed with a laser scanning microscope over time. Short video sequences of optical stacks of the GFP fluorescence covering the entire hyphae in focus were recorded after 72 h. Download Movie S3, AVI file, 18.0 MB. Copyright © 2021 Brantl et al. 2021 Brantl et al. https://creativecommons.org/licenses/by/4.0/ This content is distributed under the terms of the Creative Commons Attribution 4.0 International license.

### Inactivation of the iron acquisition repressor SreA partially compensates for loss of Asp f3 and Af3l1.

**FIG 5:**
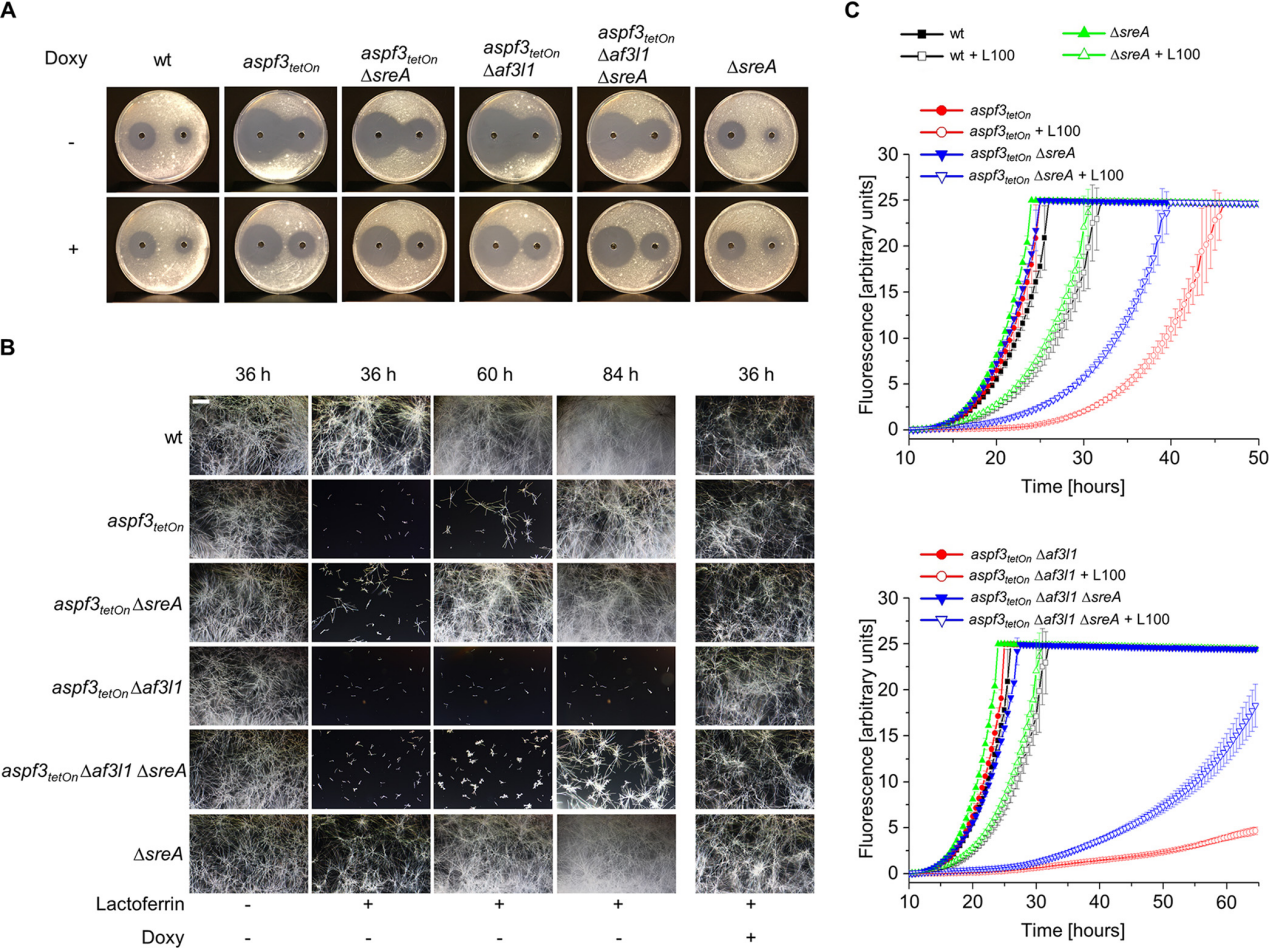
Deletion of the iron acquisition repressor *sreA* partially compensates for loss of Asp f3 and Af3l1. (A) Conidia (4 × 10^5^) of the indicated strains were spread on AMM agar plates. When indicated, medium was supplemented with doxycycline (7.5 μg ml^−1^; Doxy). Fifty microliters of 300 mM (left) or 100 mM (right) H_2_O_2_ was applied in the punch holes of each agar plate. Images were taken after 30 h of incubation at 37°C. (B and C) Conidia (1.5 × 10^3^ [B] or 1.5 × 10^4^ [C]) of the indicated strains were inoculated in RPMI 1640 medium supplemented with 100 ng ml^−1^ FeSO_4_ per well in 96-well plates. For panel C, medium was additionally supplemented with 0.002% (wt/vol) resazurin. When indicated, medium was additionally supplemented with 100 μg ml^−1^ lactoferrin (L100) or 7.5 μg ml^−1^ doxycycline (Doxy). Plates were then incubated at 37°C with 5% CO_2_. (B) After the indicated incubation time, representative dark-field images were taken. Bar, 250 μm (applicable to all images). (C) Resorufin fluorescence was documented over time with a microplate reader and plotted in the graph. The error bars indicate standard deviations for three technical replicates.

Iron uptake of A. fumigatus is under the control of a complex regulatory network. The two major regulators of iron homeostasis are SreA and HapX (28, 29). Under iron sufficiency or iron excess conditions, SreA suppresses high-affinity iron uptake and HapX triggers iron detoxification pathways. In contrast, under iron starvation, HapX activates a transcriptional response that results in repression of iron-consuming pathways and induction of iron uptake. We speculated that the low-iron growth deficiency of Aspergillus mutants lacking Asp f3 is related to incomplete derepression of iron acquisition enzymes. Deletion of *sreA* slightly improved the hydrogen peroxide tolerance of wild-type A. fumigatus and of the *aspf3_tetOn_* mutant and the *aspf3_tetOn_* Δ*af3l1* mutant under repressed conditions (Fig. 5A). The growth delay of the wild type observed under low-iron conditions was not improved upon deletion of *sreA* (Fig. 5C). In contrast, deletion of *sreA* significantly improved growth under low-iron conditions of the repressed *aspf3_tetOn_* and *aspf3_tetOn_* Δ*af3l1* mutants (Fig. 5B and C). However, it did not reach wild-type levels. This suggests either that the low-iron growth deficiency upon depletion of Asp f3 and Af3l1 is partially related to derepression of iron acquisition or that inactivation of SreA partially compensates for the defect caused by loss of peroxiredoxins indirectly by derepression of iron acquisition or by increasing the conidial iron content. In any case, these data underline a link between peroxiredoxin function and iron homeostasis.

### Asp f3 has no major impact on iron homeostasis.

**FIG 6:**
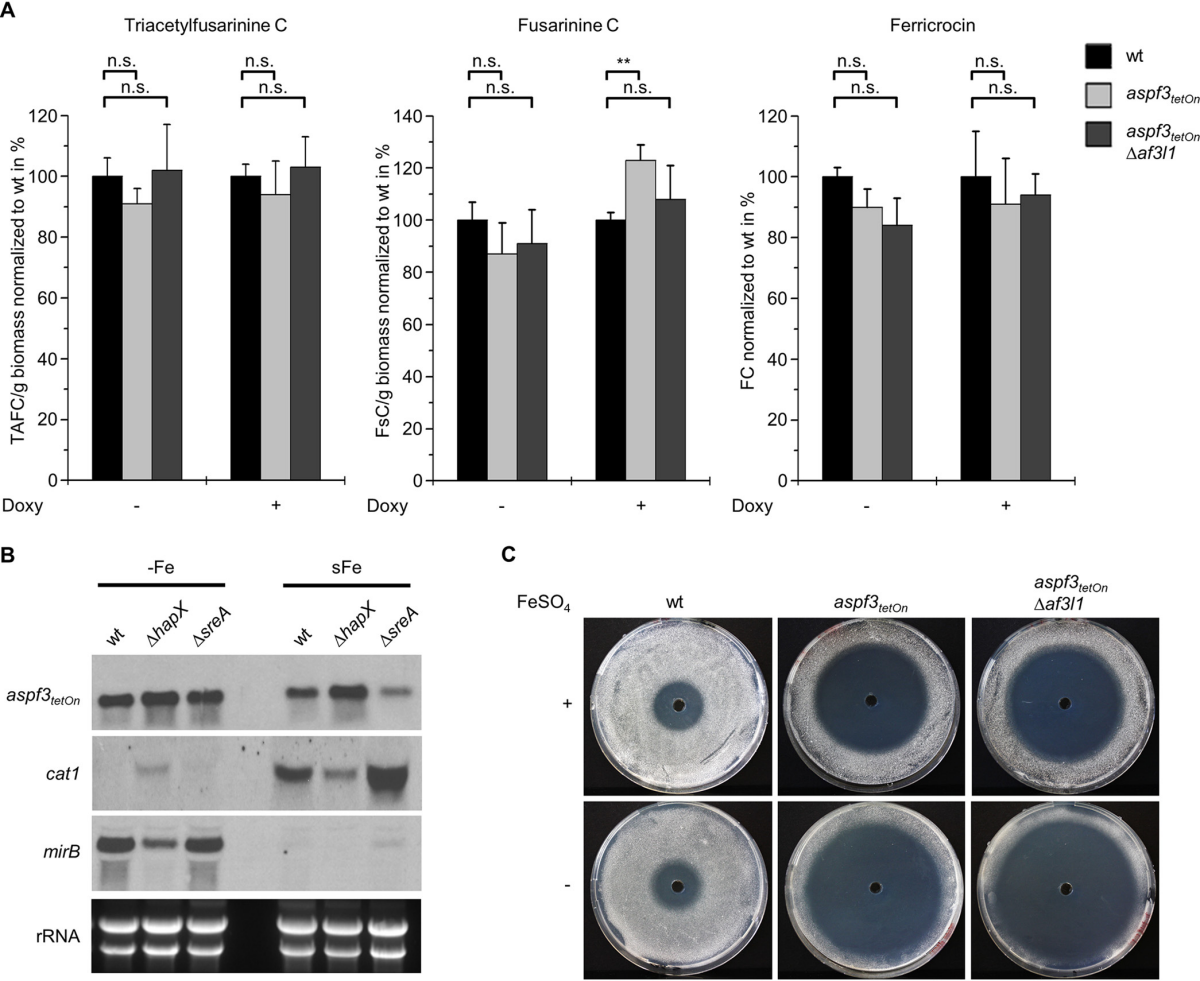
Siderophore biosynthesis is not changed in the *aspf3 af3l1* mutant, but *aspf3* expression is iron responsive. (A) Production of the extracellular (triacetylfusarinine C and fusarinine C) and intracellular (ferricrocin) siderophores in submersed growth for 18 h at 37°C in a liquid AMM variant as described in Materials and Methods was normalized to biomass and wild type (wt). When indicated, medium was supplemented with doxycycline (10 μg ml^−1^; Doxy). Statistical significance (n.s., not significant [*P* > 0.05]; **, *P* < 0.01) was calculated with one-way ANOVA with *post hoc* Tukey’s HSD test. The error bars indicate standard deviations for three technical replicates. (B) Conidia (1 × 10^6^) of A. fumigatus ATCC 46645 (wt) and of the Δ*hapX* and Δ*sreA* mutants were inoculated in 100 ml of the liquid AMM variant in shake flasks and incubated for 18 h under iron-depleted conditions at 37°C (−Fe). Subsequently, FeSO_4_ was added to a final concentration of 0.03 mM, and the cultures were incubated for one more hour at 37°C to monitor short-term adaptation from iron starvation to iron sufficiency (sFe). Total RNA was isolated before (−Fe) and after (sFe) iron addition and subjected to Northern blot analysis with *aspf3*, *cat1*, and *mirB* hybridization probes. Ethidium bromide-stained rRNA is shown as a control for RNA loading and quality. (C) Conidia (1 × 10^6^) of the indicated strains were spread on peptone agarose plates (1% [wt/vol] agarose, 1% [wt/vol] peptone; pH 7.0). When indicated, medium was supplemented with 5 μg ml^−1^ FeSO_4_ (Fe^2+^). Fifty microliters of 100 mM H_2_O_2_ was applied in the punch holes of each agar plate. Images were taken after 48 h of incubation at 37°C.

Siderophore biosynthesis is vital for growth of A. fumigatus under low-iron conditions (28, 29). We speculated that impaired growth of the *aspf3_tetOn_* Δ*af3l1* mutant under low-iron conditions could be linked to a role of the peroxiredoxins in siderophore biosynthesis regulation. We therefore analyzed production of the extracellular siderophores triacetylfusarinine C and fusarinine C and of the intracellular siderophore ferricrocin of the *aspf3_tetOn_* mutant, the *aspf3_tetOn_* Δ*af3l1* mutant, and the wild type under noninduced and induced conditions. As shown in Fig. 6A, B, and C, we found no significant differences with respect to siderophore biosynthesis in the different strains under repressed conditions. Interestingly, induction of *aspf3* expression with doxycycline seemed to result in a minor increase of fusarinine C synthesis. Nevertheless, this suggests that the growth deficiency of *aspf3_tetOn_* and *aspf3_tetOn_* Δ*af3l1* mutants under repressed conditions is not related to siderophore biosynthesis. As siderophore biosynthesis is regulated by SreA, these data also indicate that peroxiredoxins do not directly affect SreA activity (see above).

To analyze a potential direct impact of Asp f3 on iron homeostasis during germination, we analyzed transcript levels of iron-regulated genes in conidia and germinating hyphae under low-iron conditions with Northern blot analysis. Altered iron homeostasis, as seen for example in mutants that lack intracellular siderophores (e.g., a Δ*sidA* mutant), was previously shown to cause a characteristic “iron starvation signature” in the conidial transcriptome (30). As shown in Fig. S5A, *hapX* (encoding an iron-regulatory transcription factor, already mentioned above) and *ftrA* (encoding an iron permease involved in reductive iron assimilation) were not upregulated in conidia of the *aspf3_tetOn_* mutant which were obtained from mycelium on solid agar under repressed conditions. Furthermore, transcript levels of *mirB* (encoding a siderophore transporter) and *ftrA* of the *aspf3_tetOn_* mutant under repressed conditions were not significantly changed during germination under low-iron conditions (Fig. S5B). Taken together, these data indicate that Asp f3 is not a central regulator of fungal iron homeostasis.

10.1128/mBio.00976-21.5 FIG S5 (A) Conidia of the wild type, the *aspf3_tetOn_* mutant, and the Δ*sidA* mutant were harvested from cultures that were grown for 3 days at 37°C on complete medium as described in Materials and Methods without doxycycline. Harvested conidia (3 × 10^9^) were shock frozen immediately and subsequently analyzed as described below. The Δ*sidA* deletion mutant, even though in a different background strain, was included as a control. (B) Conidia (3 × 10^9^) of the wild type and the *aspf3_tetOn_* mutant were inoculated in 50 ml of a liquid AMM variant as described in Materials and Methods in shake flasks and incubated for 9 h under iron-depleted conditions at 37°C (−Fe). (A and B) Total RNA was isolated and subjected to Northern blot analysis with *hapX* and *ftrA* (A) or *ftrA* and *mirB* (B) hybridization probes. Ethidium bromide-stained rRNA is shown as a control for RNA loading and quality. Download FIG S5, PDF file, 0.06 MB. Copyright © 2021 Brantl et al. 2021 Brantl et al. https://creativecommons.org/licenses/by/4.0/ This content is distributed under the terms of the Creative Commons Attribution 4.0 International license.

### Expression of *aspf3* is responsive to iron availability.

Because of the unexpected low-iron growth phenotype of the *aspf3* mutant, we investigated whether *aspf3* expression is subject to iron regulation at the transcriptional level (Fig. 6B). The analysis of the short-term adaptation from iron starvation to iron sufficiency has previously proven to be a highly sensitive tool for identifying iron-dependent regulation (31, 32). The A. fumigatus wild type and mutants lacking either SreA or HapX, which both encode key iron regulators, were cultured under iron starvation for 18 h (−Fe). Subsequently, iron was added, and cultivation was continued for another hour (sFe). While SreA transcriptionally represses iron acquisition during iron sufficiency, HapX transcriptionally activates iron acquisition and represses iron consumption during iron starvation. Furthermore, HapX activates iron-consuming pathways and iron detoxification during short-term adaptation from iron starvation to iron sufficiency (28, 29). As strain and growth condition controls, we analyzed the transcript levels of the genes that encode the siderophore transporter MirB (*mirB*, mentioned above) and the heme iron-dependent mycelial catalase Cat1 (*cat1*). In agreement with previous reports (31, 32), *mirB* showed a decreased transcript level in the Δ*hapX* mutant during iron starvation and was significantly downregulated during short-term adaptation to iron sufficiency, with a slightly increased transcript level in the Δ*sreA* mutant. In contrast, *cat1* expression was downregulated in a HapX-dependent manner during iron starvation and induced during short-term adaptation from iron starvation to iron sufficiency. Lack of SreA caused an increased *cat1* transcript level during the short-term adaptation, which is most likely a result of increased iron acquisition and, consequently, increased activation of HapX in this strain.

During iron starvation, the wild type and the mutant strains displayed similar *aspf3* transcript levels. Upon short-term adaptation to iron sufficiency, the *aspf3* transcript level decreased slightly in the wild type and was more pronounced in the Δ*sreA* mutant, while it was largely unaffected in the Δ*hapX* mutant. The enhanced downregulation of *aspf3* during short-term adaptation to iron sufficiency in the Δ*sreA* mutant compared to the wild type could be explained by the fact that lack of SreA leads to elevated iron uptake, which would then increase the slight iron repression observed in the wild type. The lacking downregulation of *aspf3* in the Δ*hapX* mutant indicates that HapX is required for repression of *aspf3* during short-term adaptation to iron sufficiency. These data demonstrate that *aspf3* expression is modulated by iron availability at the transcript level and responds to iron availability largely inversely compared to *cat1*.

### ROS susceptibility of the repressed *aspf3_tetOn_* and *aspf3_tetOn_* Δ*af3l1* mutants is further increased under low-iron conditions.

Our data indicated that compared to the wild type, *cat1* and *aspf3* are inversely regulated in an iron- and HapX-dependent manner. The shift from iron starvation to iron sufficiency caused an increase of *cat1* expression and a decrease of *aspf3* expression. Notably, many antioxidant enzymes, such as the major catalases in A. fumigatus (Cat1 and Cat2) and the cytochrome *c* peroxidase (Ccp1), functionally depend on iron. We asked whether Asp f3 and Af3l1 are especially required under low-iron conditions to counter oxidative stress. As shown in Fig. 6C, the hydrogen peroxide inhibition zones of the wild type were indistinguishable on peptone agarose, which is low in iron, and on peptone agarose supplemented with iron sulfate. In marked contrast, the reduced availability iron on the peptone agarose medium drastically increased the hydrogen peroxide susceptibility of the *aspf3_tetOn_* and *aspf3_tetOn_* Δ*af3l1* mutants under repressed conditions.

### Iron restores virulence of the Δ*aspf3* mutant in a mammalian infection model.

**FIG 7:**
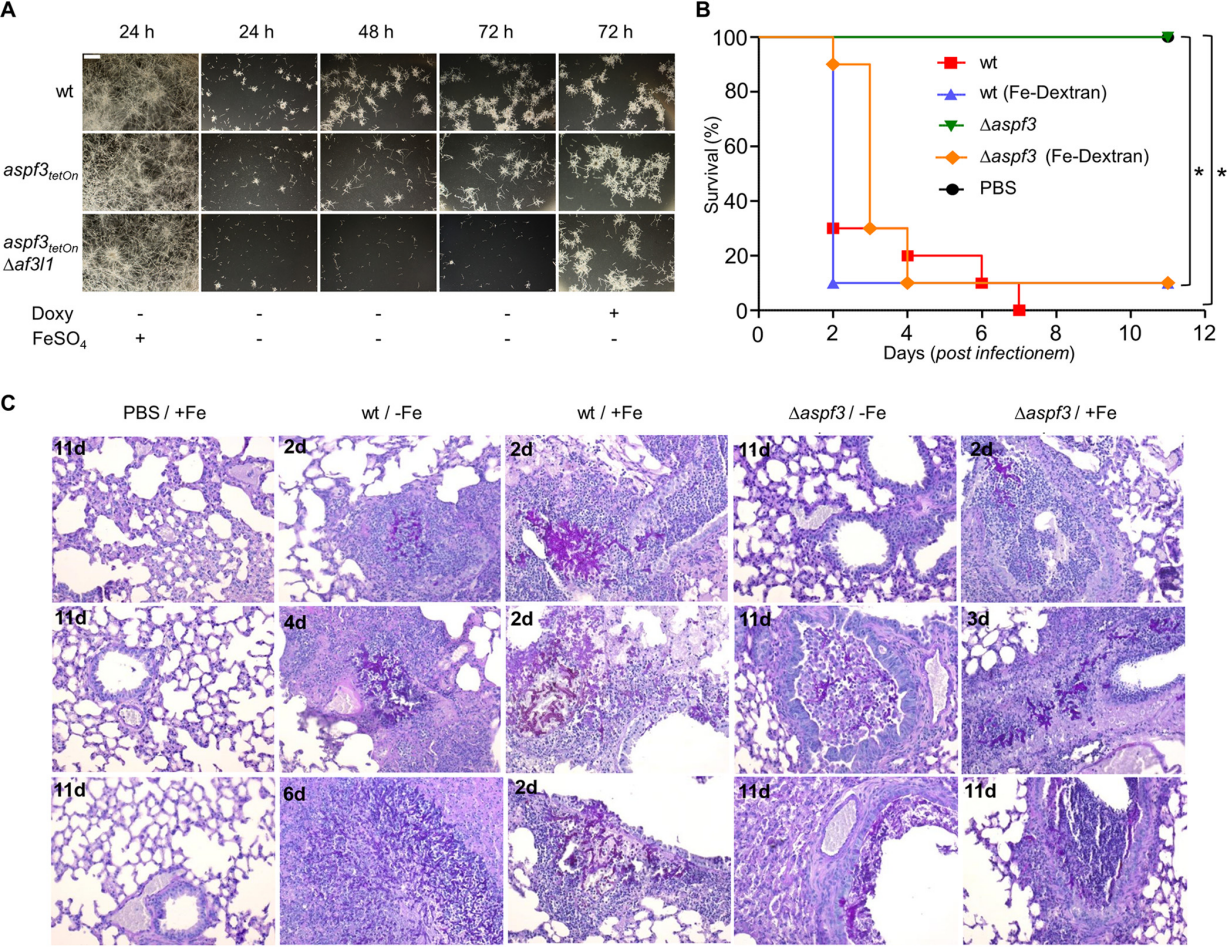
The avirulence of the Δ*aspf3* mutant is linked to the iron deprivation in the host. (A) Conidia (1.5 × 10^3^ per well) of the indicated strains were inoculated in 60% (vol/vol) serum in ddH_2_O in a 96-well plate. When indicated, medium was supplemented with 50 μg ml^−1^ FeSO_4_ or 7.5 μg ml^−1^ doxycycline (Doxy). Plates were then incubated at 37°C for the indicated times. After the indicated incubation times, representative dark-field images were taken. Bar, 250 μm (applicable to all images). (B) Survival of mice after intranasal infection with conidia of A. fumigatus D141 (wt) and the Δ*aspf3* mutant. Immunosuppressed mice were left untreated or loaded with iron (iron dextran) prior to intranasal infection with conidia (*n* = 10 per strain/treatment group). A control group (*n* = 4) was infected with PBS as a mock control. The percentage of survivors after each day of infection is shown in the graph. *, *P* < 0.001 (log-rank test and the Gehan-Wilcoxon test). (C) Histology of lungs from mice infected with A. fumigatus. Lung sections were stained with periodic acid-Schiff (PAS). The presence of tissue invasive fungal hyphae (magenta) and infiltration of immune cells (purple nuclei) was confirmed in lungs of mice infected with wild-type conidia in the presence (+Fe) or absence (−Fe) of iron and in lung sections infected with the Δ*aspf3* strain with Fe. Fungal hyphae and immune cells were rarely found in Δ*aspf3*-strain-infected lungs without Fe. Numbers in the upper left corners indicate days postinfection.

Our results demonstrated that lactoferrin specifically inhibits growth of the *aspf3_tetOn_* mutant and the *aspf3_tetOn_* Δ*af3l1* mutant under repressed conditions by sequestering available iron. This supports a model where nutritional immunity could be responsible for the previously reported avirulence of the Δ*aspf3* deletion mutant in a murine infection model (12). To further substantiate this model, we tested whether the *aspf3_tetOn_* and the *aspf3_tetOn_* Δ*af3l1* mutants have specific difficulties growing in serum. Serum is known to be a harsh environment for Aspergillus because iron is bound to transferrin and difficult to access (33, 34). As shown in Fig. 7A, growth of the *aspf3_tetOn_* mutant and, even more so, that of the *aspf3_tetOn_* Δ*af3l1* mutant in 60% (vol/vol) serum under repressed conditions was significantly reduced compared to that of the wild type. The induction of the conditional promoter with doxycycline restored growth of the two peroxiredoxin mutants to wild-type levels. In agreement with the sequestration of iron being the cause of the reduced growth of the mutants in serum, iron supplementation drastically improved growth of these strains.

This suggests that the avirulence of an Δ*aspf3* deletion mutant is primarily linked to the low-iron environment under host infection conditions. To confirm this hypothesis, we performed a murine infection experiment under conditions that overturn the low-iron conditions in the lungs of a nonneutropenic host. For this, mice were immunosuppressed with cortisone acetate, exposed to high iron levels by intraperitoneal injection of iron dextran, and infected intranasally with conidia of wild-type A. fumigatus D141 or of the corresponding Δ*aspf3* deletion mutant. For wild-type conidia, such a dose caused a lethal outcome of the infection within 2 to 7 days, independent of iron supplementation. As expected from the previous study, conidia of the Δ*aspf3* deletion mutant were avirulent. However, iron supplementation reconstituted their virulence up to wild-type levels (Fig. 7B). Iron-dependent virulence of the Δ*aspf3* strain was reflected by histopathological analysis of the lungs of infected mice (Fig. 7C). While tissue invasive hyphae and infiltration of immune cells were frequently found in the wild-type within 2 to 6 days postinfection, tissue invasion was not observed in lungs infected with the Δ*aspf3* mutant. Extensive hyphal growth of the mutant was in turn detected after iron supplementation. This shows that the avirulence of the Δ*aspf3* mutant is tightly linked to its inability to cope with low-iron conditions and demonstrates that Asp f3 is an essential key player for A. fumigatus to overcome iron limitation during infection of a host.

## DISCUSSION

Asp f3 is one of the most abundant proteins in A. fumigatus and is known for its multifaceted role as an allergen, ROS scavenger, virulence factor, and vaccine candidate (8, 10, 12–14). However, despite being studied for more than 2 decades, the cellular function of Asp f3 remains unknown. A Δ*aspf3* deletion mutant is highly susceptible to ROS. It was therefore proposed that the avirulence of the Δ*aspf3* deletion mutant in a murine infection model is attributed to more ROS-mediated damage caused by innate immune cells (12, 35, 36). However, we could not observe more efficient killing of the *aspf3_tetOn_* hyphae by human granulocytes under repressed conditions compared to wild-type hyphae. This indicates that it is not in the first instance the increased susceptibility to killing by immune cells which is the cause of the avirulence of the Δ*aspf3* deletion mutant.

In our study we discovered a new role of the peroxiredoxin Asp f3 and its homologue Af3l1, which they redundantly exert. The Δ*aspf3* deletion mutant and the conditional *aspf3_tetOn_* mutant under repressed conditions have severe problems growing under iron-limited conditions. Additional deletion of *af3l1* significantly reinforces this growth defect. A second Asp f3 homologue, Af3l2, appears not to be involved in iron homeostasis, as the deletion of the encoding gene did not reinforce the investigated phenotype. This is in agreement with the finding that Af3l2, in contrast to Asp f3 and Af3l1, harbors a putative N-terminal mitochondrial targeting signal which presumably guides this protein to a subcellular compartment distinct from that of Asp f3 and Af3l1.

Homologues of Asp f3 and Af3l1 are found in other fungal pathogens. Close homologues in A. nidulans (AnPrxA), C. albicans (CaAhp1), and S. cerevisiae (ScAhp1) were shown to have a role in detoxification of ROS, similar to what was previously described for A. fumigatus Asp f3 (12, 25, 37, 38). To our knowledge, neither Asp f3 nor any of its homologues in other species has been linked to iron homeostasis or growth under iron-limited conditions. We speculated that Asp f3 and Af3l1 could be involved in iron homeostasis or siderophore biosynthesis. However, the expression of genes which are typically regulated dependent on the intracellular iron availability, such as those encoding the iron-regulatory transcription factor HapX, the iron permease FtrA, and the siderophore transporter MirB, were not significantly altered in the *aspf3_tetOn_* mutant under repressed and low-iron conditions compared to the wild type. Moreover, biosynthesis of the extracellular siderophores triacetylfusarinine C and fusarinine C and of the intracellular siderophore ferricrocin was not significantly changed in mutants lacking Asp f3 and Af3l1. These results strongly argue against a key regulatory role of Asp f3 and Af3l1 in maintaining iron homeostasis of A. fumigatus under low-iron conditions.

Interestingly, deletion of *sreA*, a gene which encodes the important iron acquisition repressor SreA, partially compensates for the growth defect of the repressed *aspf3_tetOn_* and *aspf3_tetOn_* Δ*af3l1* mutants under iron-limited conditions. Surprisingly, ROS tolerance of the *aspf3_tetOn_* and *aspf3_tetOn_* Δ*af3l1* mutants under repressed conditions was also slightly improved upon deletion of *sreA*. The only partial compensations indicate that the low-iron growth deficiency and the ROS susceptibility are not mediated via SreA hyperactivity in the repressed *aspf3_tetOn_* and *aspf3_tetOn_* Δ*af3l1* mutants. This is underlined by the fact that siderophore production, which is regulated by SreA (31), was wild type-like in *aspf3_tetOn_* and *aspf3_tetOn_* Δ*af3l1* mutants. Congruently, it suggests that the ROS susceptibility and the low-iron growth deficiency are linked, as SreA seemingly affects both.

Our data demonstrate that iron depletion with lactoferrin remarkably inhibits germination of A. fumigatus conidia which lack Asp f3. Even though lactoferrin was reported to have multiple antimicrobial activities (39), the specific susceptibility of the *aspf3* mutant found in this study is unambiguously linked to the iron sequestration. First, other iron-depleting conditions, e.g., adding the iron chelator BPS, caused a very similar growth defect. Second, supplementation of the lactoferrin-containing medium with iron fully suppressed the growth defect. Lactoferrin is found in excessive amounts in many body fluids, including lung secretions, where it serves as an antimicrobial peptide and sequesters iron (40, 41).

An adequate supply of essential trace elements is crucial for fungal and bacterial pathogens to establish an infection (28, 42). Because of this, metazoa evolved multiple mechanisms to deprive possibly invading microbes from essential trace elements such as iron, a concept which is called nutritional immunity (43). Our results demonstrate that Asp f3 and its homologue Af3l1 are essentially required to grow and to survive under iron-limited conditions. Importantly, we could show that Asp f3 and Af3l1 are required to overcome the iron limitation in human serum. Furthermore, we could show that supplementation with iron restores the tissue invasive growth and the virulence of the Δ*aspf3* deletion mutant even in the nonneutropenic murine infection model used here. Together, this indicates that the avirulence of the Δ*aspf3* deletion is primarily caused by the inability of the mutant to grow under iron-limited conditions, rather than resulting from an increased susceptibility to innate immune cells. It should not be ruled out at this point that *in vivo* innate immune cells, such as neutrophilic granulocytes, may additionally limit the access to iron, e.g., via the well-documented release of lactoferrin (44), or in addition may more efficiently inactivate the mutant because of the increased ROS susceptibility under iron-deprived conditions. In agreement with this model, *aspf3* expression is regulated in a HapX- and iron availability-dependent manner. This makes Asp f3 an important virulence factor which is specifically required to overcome iron-depriving nutritional immunity during infection of the host. In summary, this study highlights for the first time the crucial role of a peroxiredoxin as a virulence factor which is specifically required to overcome iron-depriving nutritional immunity during infection of the host.

What is the mechanism by which Asp f3 and Af3l1 enable the mold to grow and survive under iron-limited conditions? A recent study performed with baker’s yeast revealed that yeast cells that starve produce nontoxic levels of ROS, which results in the unconventional secretion of normally intracellular antioxidant enzymes, including the Asp f3/Af3l1 homologue ScAhp1 (45). The authors concluded that there is a mechanism whereby antioxidants maintain the cells in a form necessary for growth in case they later return to normal conditions. Interestingly, Asp f3 was also found to be secreted despite lacking a conventional secretion signal in several independent studies (14, 46–48). It is conceivable that A. fumigatus and other fungi also rely on antioxidant enzymes to constrain their ROS formation under stressful conditions, such as iron deprivation or germination (49–51). We propose that Asp f3 and Af3l1 play a major role here. The main antioxidant enzymes, e.g., Cat1 (mycelial catalase), Cat2 (catalase-peroxidase), and Ccp1 (cytochrome *c* peroxidase), all functionally depend on iron, an essential constituent of their heme groups, and are consequently transcriptionally downregulated during iron starvation to spare iron and to prevent futile protein synthesis (31, 32, 49). Since peroxiredoxins do not depend on heme, Asp f3 and Af3l1 can step in and could allow the mold to overcome the stress conditions during iron deprivation. In line with this model, we found that (i) even the hyphae of the iron-deprived *aspf3_tetOn_* Δ*af3l1* mutant under repressed conditions stay alive for several days, reminiscent of the starving yeast cells which depend on antioxidant enzymes to continue to grow (45); (ii) the transcription of *cat1* and that of *aspf3* are inversely regulated depending on iron availability; and (iii) the susceptibility of the repressed *aspf3_tetOn_* and *aspf3_tetOn_* Δ*af3l1* mutants to hydrogen peroxide increases even further under low-iron conditions. Future studies will have to explore the cellular function of these peroxiredoxins during iron deprivation in further detail.

## MATERIALS AND METHODS

### Strains, culture conditions, and chemicals.

The strains used and constructed in this study are listed in Table 1. The strains used in this study are derivatives of the A. fumigatus strain D141. The Δ*aspf3* deletion mutant is a direct derivative of D141 and was described previously (12). The D141 derivative AfS35 is a nonhomologous end joining-deficient strain (52, 53) and was used as progenitor (wild type [wt]) for all mutants constructed in this study. The conditional *aspf3_tetOn_* mutant was constructed as described before (54). Briefly, the doxycycline-inducible *oliC*-*tetOn* promoter cassette derived from pJW128 was inserted before the coding sequence of *aspf3* by double-crossover homologous recombination. Deletion mutants were constructed by replacing the coding region of the respective genes with a self-excising hygromycin B resistance cassette that was obtained from pSK528, essentially as described before (53, 55). Mitochondria were visualized with mitochondrion-targeted green fluorescent protein (mtGFP). To this end, strains were transformed with pCH005, which expresses an N-terminal mitochondrial targeting signal fused to a GFP derivative (sGFP), essentially as described before (56).

**TABLE 1:** A. fumigatus strains used in this work

Strain or genotype	Relevant genetic modification	Parental strain	Reference
D141			66
Δ*aspf3*	*aspf3*::*hygro*^r^	D141	12
AfS35 (wt, if not stated differently)	*akuA*::*loxP*	D141	52
*aspf3_tetOn_*	*aspf3(p)*::*ptrA-tetOn*	AfS35	This work
Δ*af3l1*	*af3l1*::*six-xylP-*β-*rec-trpCt-ptrA-six*	AfS35	This work
Δ*af3l2*	*af3l2*::*six-xylP-*β-*rec-trpCt-ptrA-six*	AfS35	This work
Δ*af3l1* Δ*afl2*	*af3l1*::*six af3l2*::*six-xylP-*β-*rec-trpCt-ptrA-six*	Δ*af3l1*	This work
*aspf3_tetOn_* Δ*af3l1*	*af3l1*::*six-xylP-*β-*rec-trpCt-ptrA-six*	*aspf3_tetOn_*	This work
*aspf3_tetOn_* Δ*af3l2*	*af3l2*::*six-xylP-*β-*rec-trpCt-ptrA-six*	*aspf3_tetOn_*	This work
Δ*af3l1* Δ*af3l2 aspf3_tetOn_*	*aspf3(p)*::*ptrA-tetOn*	Δ*af3l1* Δ*afl2*	This work
Δ*sreA*	*sreA*::*six-xylP-*β-*rec-trpCt-ptrA-six*	AfS35	This work
*aspf3_tetOn_* Δ*sreA*	*sreA*::*six-xylP-*β-*rec-trpCt-ptrA-six*	*aspf3_tetOn_*	This work
*aspf3_tetOn_* Δ*af3l1* Δ*sreA*	*af3l1*::*six sreA*::*six-xylP-*β-*rec-trpCt-ptrA-six*	*aspf3_tetOn_* Δ*af3l1*	This work
wt + mtGFP	pCH005	AfS35	15
*aspf3_tetOn_*+ mtGFP	pCH005	*aspf3_tetOn_*	This work
*aspf3_tetOn_* Δ*af3l1 *+ mtGFP	pCH005	*aspf3_tetOn_* Δ*af3l1*	This work
ATCC 46645			31
Δ*sreA*	*sreA*::*hygro*^r^	ATCC 46645	31
Δ*hapX*	*hapX*::*hygro*^r^	ATCC 46645	32
Δ*sidA*	*sidA*::*hygro*^r^	ATCC 46645	67

Strains were cultured on Aspergillus minimal medium (AMM) (57) to harvest conidia if not stated differently. Experiments were performed on or in AMM, RPMI 1640 medium (11835-063; Gibco, Thermo Fisher, Waltham, MA), or peptone medium (pH 7.0) (LP0034B; Oxoid, Thermo Fisher Scientific, Rockford, IL, USA). If not stated differently, experiments performed in RPMI 1640 included incubation with 5% CO_2_ and solid media were supplemented with 2% (wt/vol) agar (214030; BD Bioscience, Heidelberg, Germany) or agarose (141098; Serva, Heidelberg, Germany). Resazurin (R7017), paraformaldehyde (158127), bathophenanthrolinedisulfonic acid (BPS; 146617) and calcofluor white (F3543) were obtained from Sigma-Aldrich (St. Louis, MO, USA), hydrogen peroxide (H_2_O_2_; 8070.2), EDTA (8040.1) and FeSO_4_ (P015.1) were obtained from Carl Roth (Karlsruhe, Germany), doxycycline was obtained from Clontech (631311; Mountain View, CA, USA), and Percoll was obtained from GE Healthcare (10253000; Uppsala, Sweden). Lactoferrin from bovine milk was purchased from Ingredia (PEP10LAC02; Ingredia SA, Arras Cedex, France).

### MitoFLARE assay with human granulocytes and growth experiment in serum.

Viability of Aspergillus hyphae after exposure to human granulocytes was analyzed as previously described (15). Briefly, 3 × 10^3^ conidia of the indicated strains that express mtGFP were inoculated in 300 μl RPMI 1640 per well in μ-Slide 8-well slides (number 80826; Ibidi, Martinsried, Germany). Slides were incubated at 37°C with 5% CO_2_. After 10 h of incubation, 1.5 × 10^6^ granulocytes resuspended in 100 μl RPMI 1640 were added per well. After the indicated incubation time, samples were fixed with 4% (wt/vol) paraformaldehyde for 10 min followed by staining with calcofluor white (1 mg ml^−1^ in double-distilled water [ddH_2_O]) for 10 min. Samples were subsequently washed with phosphate-buffered saline (PBS). In each experiment, 60 hyphae per sample and three samples per condition were analyzed using a fluorescence microscope and a 63× objective with oil immersion by an assessor who was blind to sample identity. Samples with different strains were randomly located in each μ-Slide 8-well slide. The calcofluor white and GFP fluorescence and the mitochondrial morphology were analyzed as described before (15). Prior to the blind analysis of an experiment, the killing efficacy of each batch of isolated granulocytes was evaluated with a nonblind wild-type control sample. Experiments where excessive or no significant killing was observed were excluded and not considered in the subsequent statistical analysis (hyphal vitality of wild type after killing for 2 h of <30% or >90%). On that score, of four experiments, one (25%) was excluded because of too-high killing activity and no experiment (0%) because of too-low killing activity. Statistical significance was calculated with a two-tailed unpaired (assuming unequal variances) Student's *t* test posttest. Statistical analysis was done with GraphPad Prism 5 (GraphPad Software, La Jolla, CA, USA).

Granulocytes and serum for the growth inhibition experiment were isolated from the blood of healthy adult volunteers. For serum outgrowth experiments, 1.5 × 10^3^ conidia of the indicated strains were inoculated in 60% (vol/vol) serum in ddH_2_O per well in a 96-well plate. The plates were incubated at 37°C for the indicated time. The method for isolating the granulocytes was described before (15). Volunteers gave informed written consent; collection was conducted according to the Declaration of Helsinki and was approved by the Ethics Committee of the LMU München.

### Analysis of metabolic activity.

The metabolic activity of Aspergillus hyphae was analyzed over time with a resazurin reduction assay (58). Conidia (1.5 × 10^4^) were inoculated in 200 μl RPMI 1640 supplemented with 0.002% (wt/vol) resazurin per well in a 96-well plate (Z707902; TPP, Sigma-Aldrich). Strains were inoculated in triplicate for each experiment. When indicated, medium was additionally supplemented with lactoferrin or FeSO_4_. Well plates were sealed with a Breathe-Easy sealing membrane (Z380059; Sigma-Aldrich) and incubated. Plates were subsequently incubated at 37°C with 5% CO_2_ and analyzed over time in a BMG Labtech CLARIOstar microplate reader (excitation, 550-15 nm; dichroic mirror, 568.8 nm; emission, 590-20 nm; excitation and detection from the top; BMG Labtech, Ortenberg, Germany).

### Microscopy.

Fluorescence microscopy to obtain images or to analyze mitochondrial dynamics was performed with a Leica SP5 inverted confocal laser scanning microscope (Leica Microsystems, Mannheim, Germany) equipped with a climate chamber (The Cube & The Box, Life Imaging Services, Switzerland). Quantitative analysis of the killing activity of human granulocytes against Aspergillus hyphae was performed with a Leica DM IRB inverted microscope (Leica Microsystems). If not stated differently, 3 × 10^3^ conidia were inoculated in 300 μl medium per well in a μ-Slide 8-well slide for fluorescence microscopy. Conventional bright- and dark-field images were taken with an EOS 550D digital camera (Canon, Tokyo, Japan) fitted to an Axiovert 25 inverted microscope (Carl Zeiss MicroImaging, Göttingen, Germany).

### Bioinformatics.

Sequences were obtained from FungiDB (59), the *Saccharomyces* Genome Database (60), and the *Candida* Genome Database (61). Alignments were performed with Jalview (62) and MAFFT (multiple alignment using fast Fourier transform); the average distance tree was generated with BLOSUM62.

### Murine infection experiment.

Prior to infection, A. fumigatus D141 and the Δ*aspf3* mutant were grown on malt peptone (MP) agar with 19 g liter^−1^ malt extract broth (CP75.1; Carl Roth) and 15 g liter^−1^ agar (5210.2; Carl Roth) with or without 1% (wt/vol) iron dextran (Mediferran; Medistar; 200 mg/ml iron[II] ion) at 37°C for 5 days. Conidia were harvested with sterile PBS with 0.01% (vol/vol) Tween 20 with or without 2% (wt/vol) iron dextran. Conidia were quantified using an automatic cell counter (CASY, model TT; OLS OMNI Life Science, Bremen, Germany). Specific-pathogen-free female outbred CD-1 mice (18 to 20 g; 6 to 8 weeks old) were obtained from Charles River, Germany. Animals were housed under standard conditions in individually ventilated cages and fed normal mouse chow and water *ad libitum*. All animals were cared for in accordance with the European animal welfare regulation, and animal experiments were approved by the responsible federal/state authority and ethics committee in accordance with the German animal welfare act (permit no. 03-009/15). Mice were immunosuppressed with two single doses of 1 g cortisone acetate (C3130-25G, Sigma-Aldrich) per kg of body weight, which were injected intraperitoneally 3 days before and immediately prior to infection with conidia (day 0). Iron loading of mice was achieved by intraperitoneal (i.p.) injection of 20 mg of iron dextran. Subsequently, mice were anesthetized by an intraperitoneal anesthetic combination of midazolam, fentanyl, and medetomidine. Conidia (1 × 10^6^) in 20 μl PBS were applied to the nares of the mice. Deep anesthesia ensured inhalation of the conidial inocula. Anesthesia was terminated by subcutaneous injection of flumazenil, naloxone, and atipamezole. Infected animals were monitored twice daily to check weight loss and dyspnea. Analysis of the survival data for the murine infection model was assessed with the log-rank test and the Gehan-Wilcoxon test, and *P* values of <0.05 were considered significant.

### Histopathological analysis.

Lungs of sacrificed mice were fixed in buffered formalin and embedded in paraffin. Sections of 4 μm were deparaffinized, hydrated in water, and stained with periodic acid-Schiff stain (PAS) using standard protocols. Briefly, sections were oxidized by staining with 1% PA solution for 5 min, incubated with Schiff’s reagent for 15 min, and rinsed with tap water between treatments. Counterstaining was carried out with hematoxylin for 30 s, followed by thorough washes with tap water. Stained sections were visualized by light microscopy.

### Quantitative analysis of siderophore biosynthesis.

For analysis of siderophore production, A. fumigatus wild-type and mutant strains were grown for 18 h at 37°C in a liquid Aspergillus minimal medium variant (63) with 20 mM glutamine as the nitrogen source and 1% glucose as the carbon source, omitting addition of iron to generate iron starvation conditions using 10^6^ conidia ml^−1^ for inoculation. Production of secreted triacetylfusarinine C and fusarinine C as well of intracellular ferricrocin was quantified from culture supernatants and cell extracts as described previously (64). Produced fungal biomass was harvested by filtration and weighted after freeze-drying for normalization of siderophore production to biomass. Experiments were carried out in triplicate. Statistical significance was calculated with one-way analysis of variance (ANOVA) with a *post hoc* Tukey’s honestly significant difference (HSD) test calculator (https://astatsa.com/).

### Northern blot analysis.

To analyze hyphae, conidia of the respective strains were cultured in a liquid Aspergillus minimal medium variant (63) with 20 mM glutamine as the nitrogen source and 1% glucose as the carbon source, omitting addition of iron to generate iron starvation conditions. Complete medium (2% [wt/vol] glucose, 0.2% [wt/vol] peptone, 0.1% [wt/vol] yeast extract, 0.1% [wt/vol] Casamino Acids, 7 mM KCl, 2 mM MgSO_4_, 11 mM KH_2_PO_4_, and trace elements as described in the recipe for AMM but without iron, with pH adjusted with HCl to 6.5) was used to obtain conidia for RNA isolation and Northern blot analysis. RNA isolation and Northern blot analysis, using 10 μg of extracted RNA, were performed essentially as described previously (65). The digoxigenin-labeled hybridization probes used in this study were generated by PCR. Primers used were 5′-ATGTCTGGACTCAAGGCCG and 5′-TTACAGGTGCTTGAGGACGG for *aspf3*, 5′-CCAATGCGGTATGTCCCT and 5′-GAGTCATGAGCAGTGGCA for *cat1*, 5′-AAGCCGAGAAAAAGGGGG and 5′-AACCCAGATGAAGCCCAG for *mirB*, 5′-ATGGCAAAAGACGTATTTGC and 5′-TCAGACAAGGGATGCTC for *ftrA*, and 5′-TCGGTGGAAAGAAGTGCC and 5′-CGAGTCCGTTTGGGTATC for *hapX*.
