# Supplementary figures and images for: Peroxiredoxin Asp f3 Is Essential for Aspergillus fumigatus To Overcome Iron Limitation during Infection
Source: mBio. 2021 Aug 17;12(4):e00976-21. doi: 10.1128/mBio.00976-21 (PMC8406167; doi:10.1128/mBio.00976-21)

Supplementary fig. 1

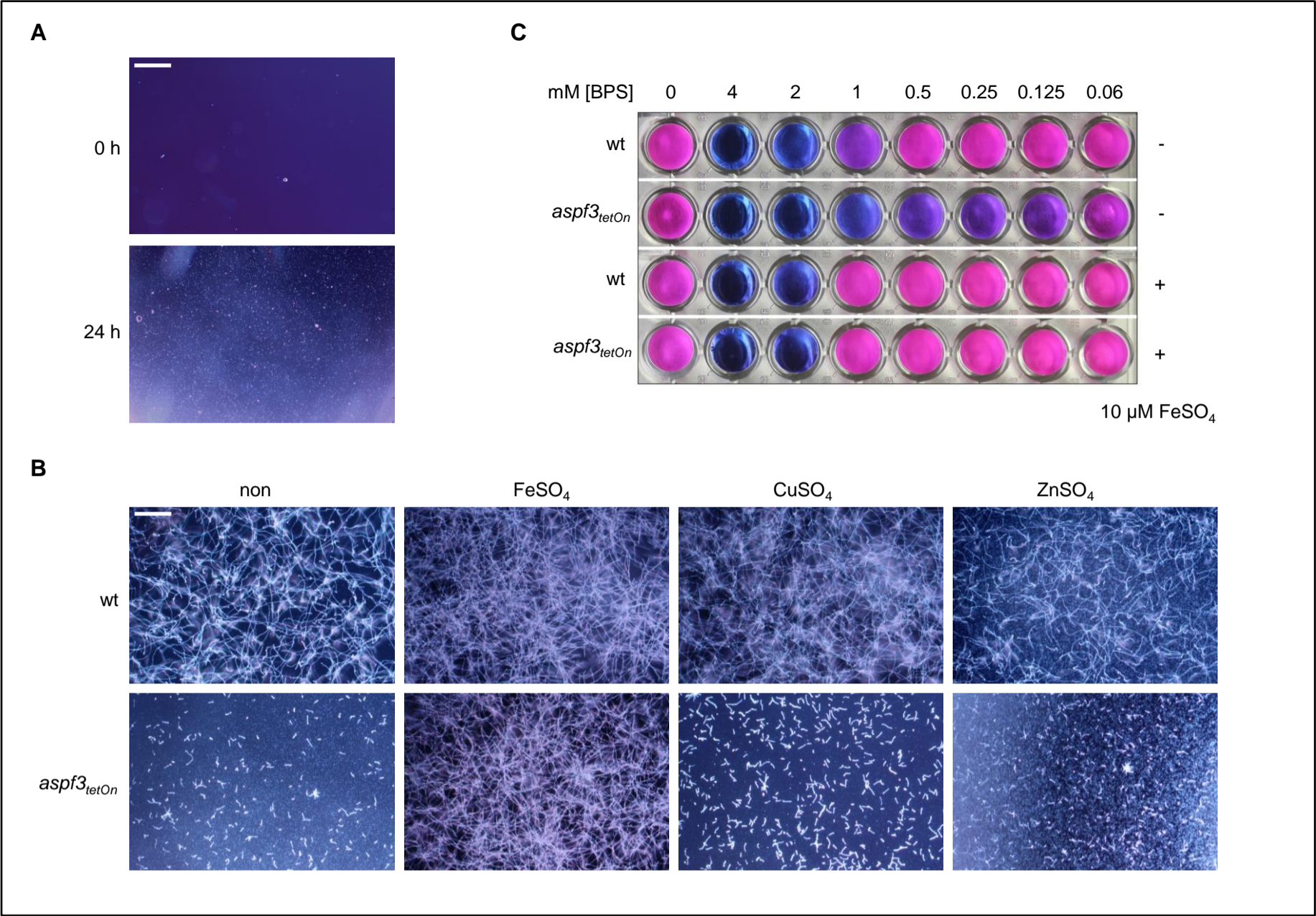

Supplement: FIG S1 [file mbio.00976-21-sf001.pdf]

Supplementary fig. 2

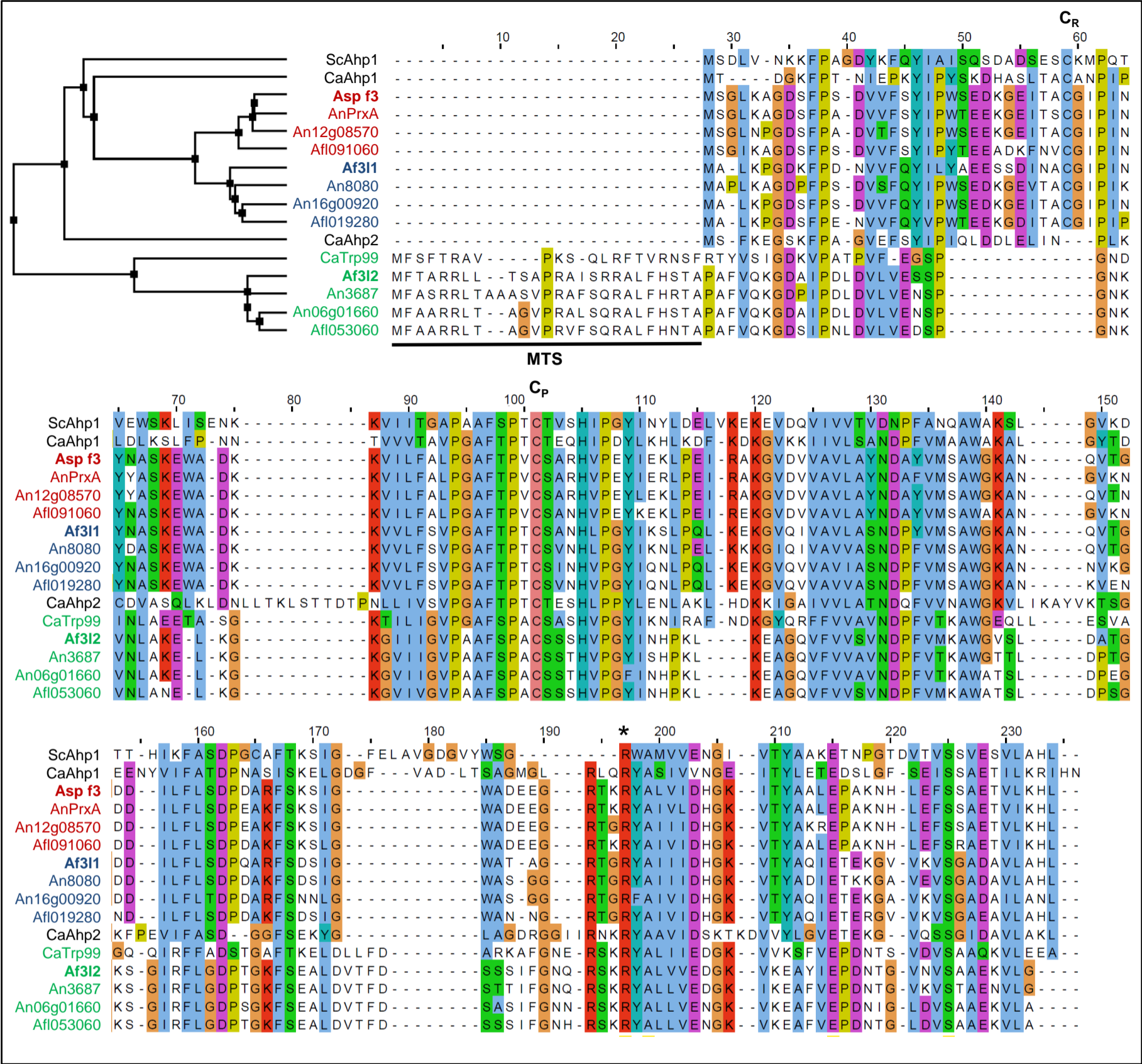

Supplement: FIG S2 [file mbio.00976-21-sf002.pdf]

Supplementary fig. 3

A

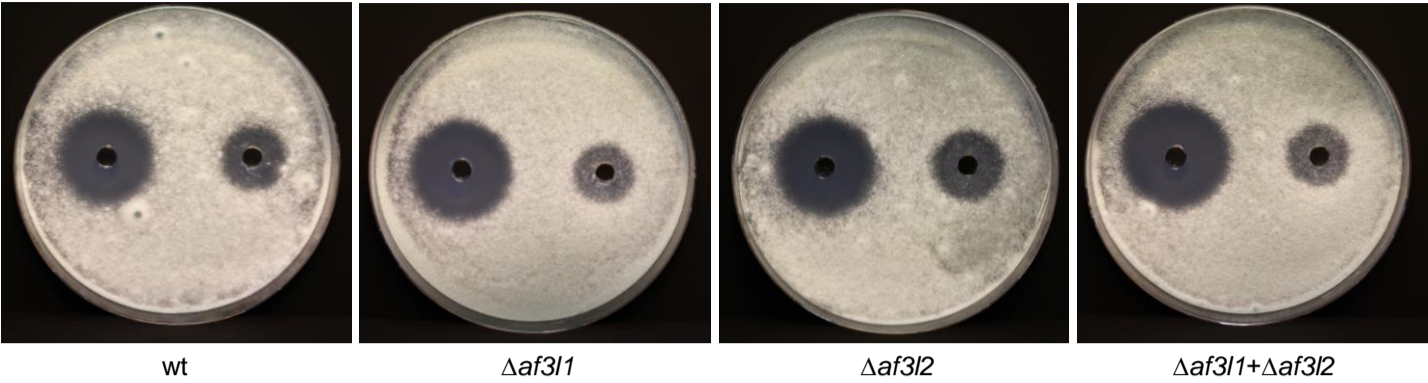

B

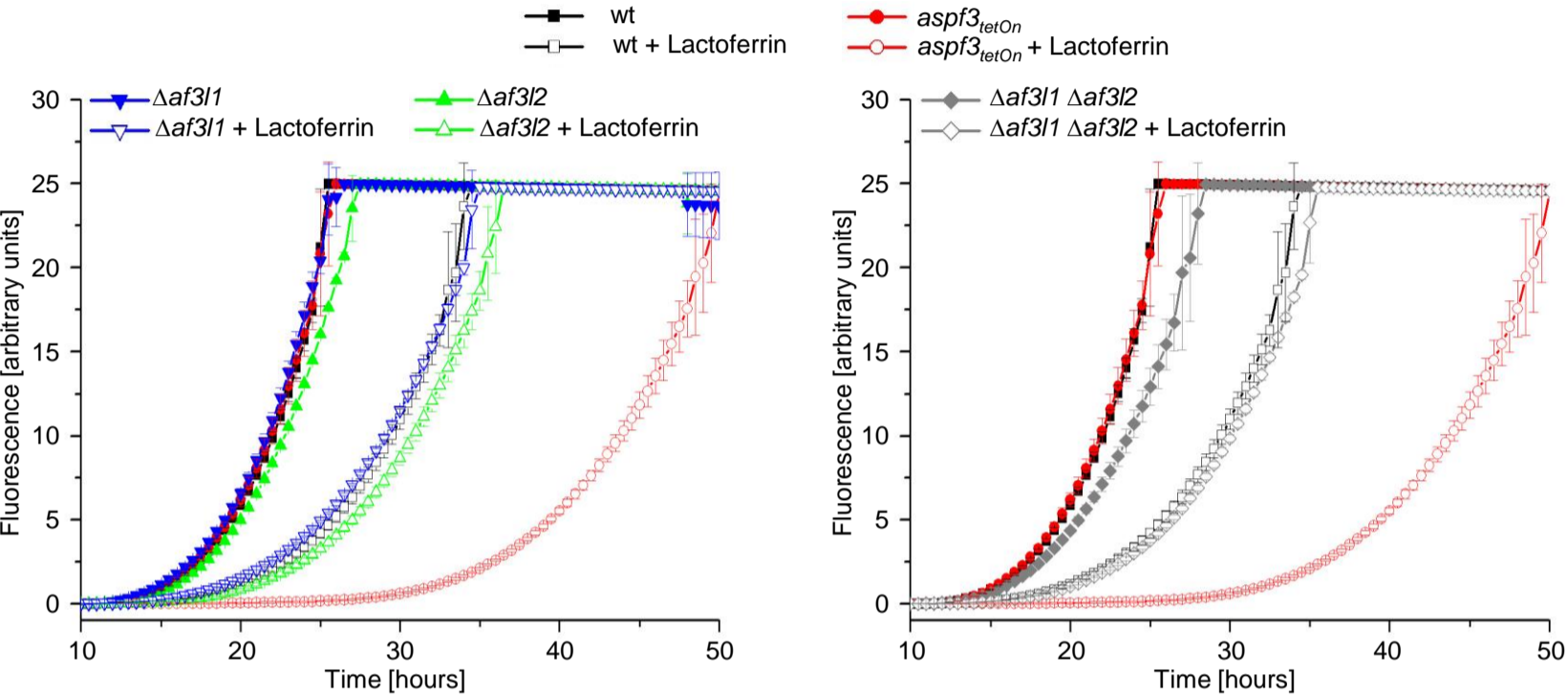

Supplement: FIG S3 [file mbio.00976-21-sf003.pdf]

Supplementary fig. 4

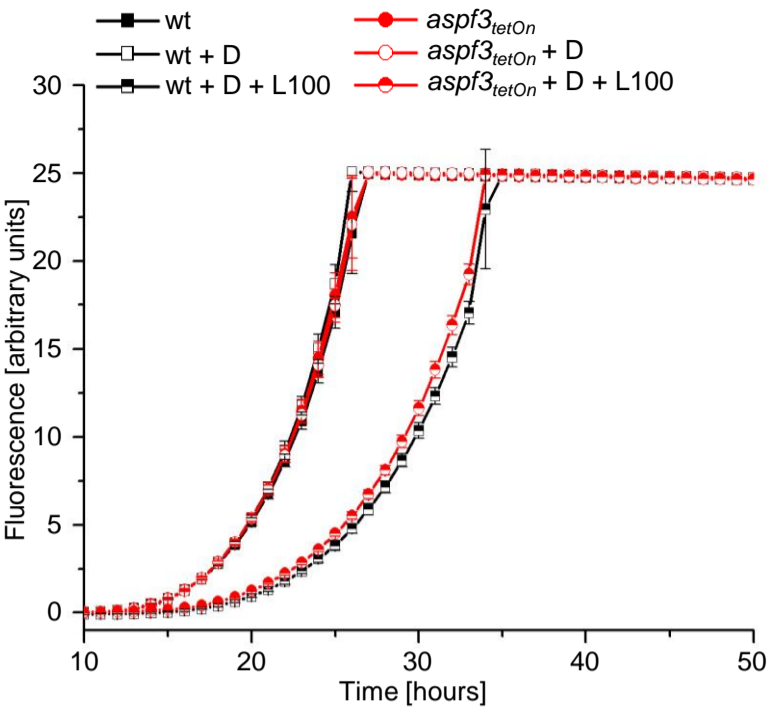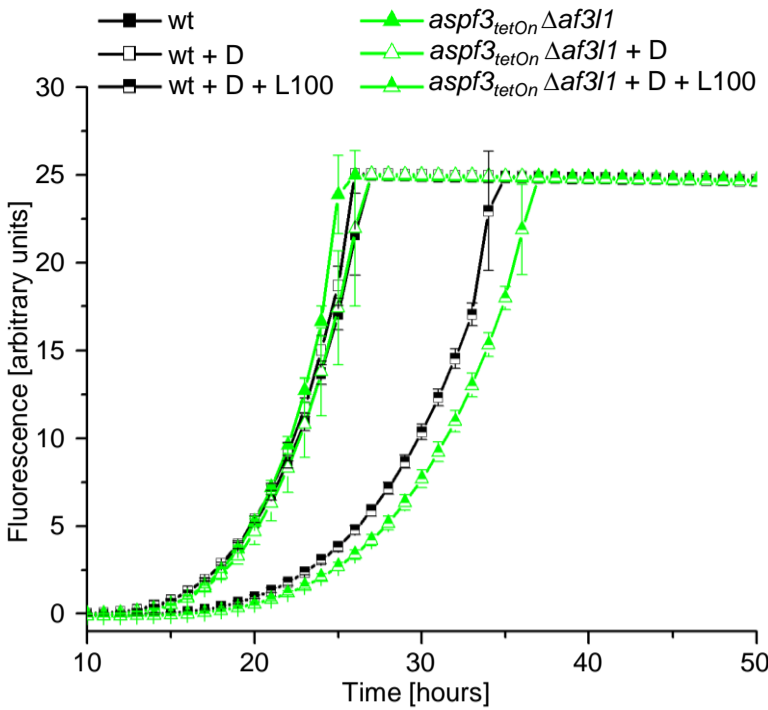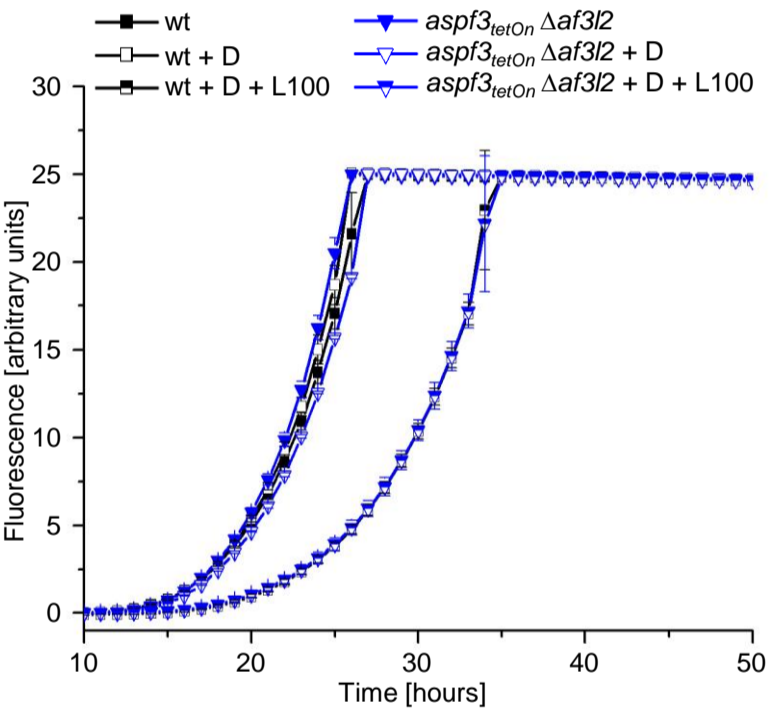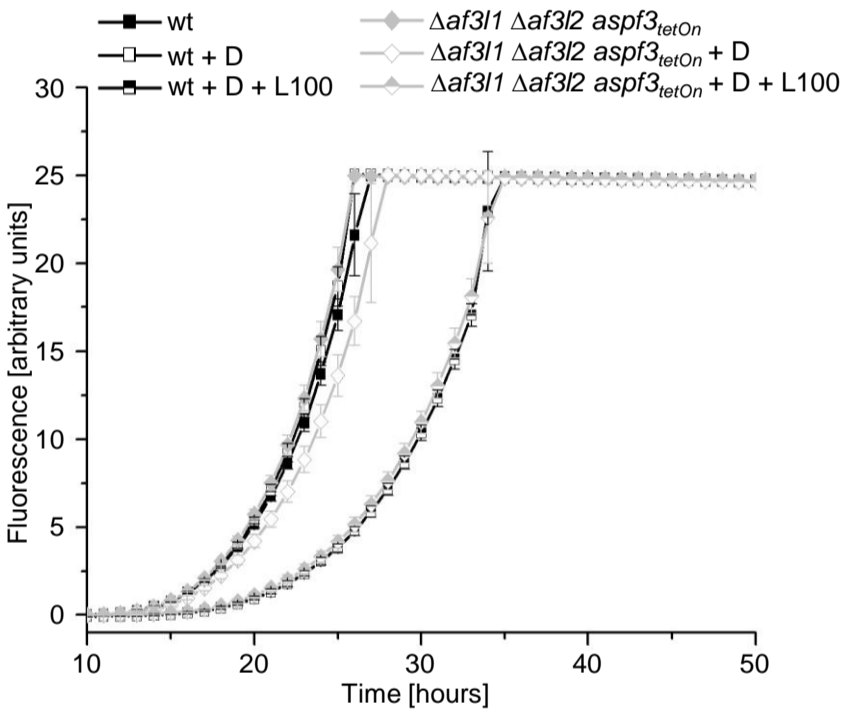

Supplement: FIG S4 [file mbio.00976-21-sf004.pdf]

Supplementary figure 5

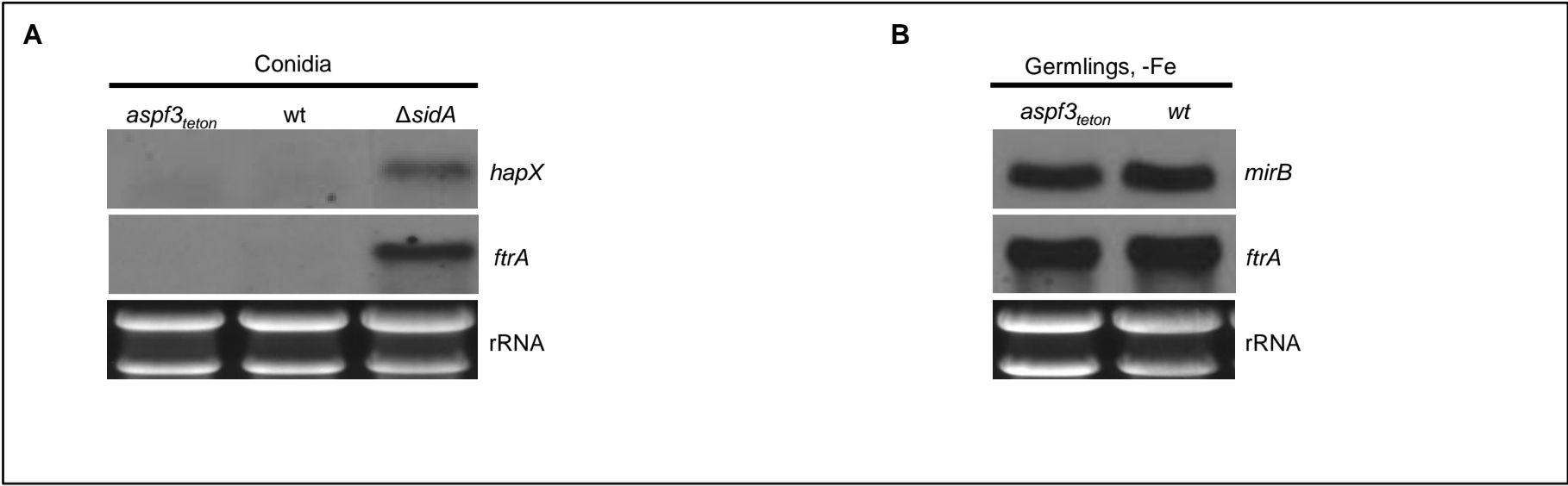

Supplement: FIG S5 [file mbio.00976-21-sf005.pdf]
